# Vibrational kinetics in repetitively pulsed atmospheric pressure nitrogen discharges: average-power-dependent switching behaviour

**DOI:** 10.1088/1361-6595/aca9f4

**Published:** 2023-02-08

**Authors:** Helen L Davies, Vasco Guerra, Marjan van der Woude, Timo Gans, Deborah O’Connell, Andrew R Gibson

**Affiliations:** 1 York Plasma Institute, Department of Physics, University of York, Heslington, YO10 5DD, United Kingdom; 2 Instituto de Plasmas e Fusão Nuclear, Instituto Superior Técnico, Universidade de Lisboa, 1049-001 Lisboa, Portugal; 3 York Biomedical Research Institute and Hull York Medical School, University of York, Heslington, YO10 5DD, United Kingdom; 4 School of Physical Sciences, National Centre for Plasma Science and Technology, Dublin City University, Dublin 9, Ireland; 5 Research Group for Biomedical Plasma Technology, Ruhr-Universität Bochum, Universitätsstraße 150, 44801 Bochum, Germany

**Keywords:** vibrational state kinetics, dielectric barrier discharge, vibrational up-pumping, atmospheric pressure plasmas

## Abstract

Characterisation of the vibrational kinetics in nitrogen-based plasmas at atmospheric pressure is crucial for understanding the wider plasma chemistry, which is important for a variety of biomedical, agricultural and chemical processing applications. In this study, a 0-dimensional plasma chemical-kinetics model has been used to investigate vibrational kinetics in repetitively pulsed, atmospheric pressure plasmas operating in pure nitrogen, under application-relevant conditions (average plasma powers of 0.23–4.50 W, frequencies of 1–10 kHz, and peak pulse powers of 23–450 W). Simulations predict that vibrationally excited state production is dominated by electron-impact processes at lower average plasma powers. When the average plasma power increases beyond a certain limit, due to increased pulse frequency or peak pulse power, there is a switch in behaviour, and production of vibrationally excited states becomes dominated by vibrational energy transfer processes (vibration–vibration (V–V) and vibration–translation (V–T) reactions). At this point, the population of vibrational levels up to $v\,\leqslant\,40$ increases significantly, as a result of V–V reactions causing vibrational up-pumping. At average plasma powers close to where the switching behaviour occurs, there is potential to control the energy efficiency of vibrational state production, as small increases in energy deposition result in large increases in vibrational state densities. Subsequent pathways analysis reveals that energy in the vibrational states can also influence the wider reaction chemistry through vibrational–electronic (V–E) linking reactions (N + N$_2(40\,\leqslant\,v\,\leqslant\,45) \rightarrow $ N$(^2D)$ + N$_2(A)$ and N + N$_2(39\,\leqslant\,v\,\leqslant\,45) \rightarrow$ N + N$_2(a^{\prime})$), which result in increased Penning ionisation and an increased average electron density. Overall, this study investigates the potential for delineating the processes by which electronically and vibrationally excited species are produced in nitrogen plasmas. Therefore, potential routes by which nitrogen-containing plasma sources could be tailored, both in terms of chemical composition and energy efficiency, are highlighted.

## 1. Introduction

Low temperature plasmas (LTPs) using nitrogen or nitrogen mixtures as a feed gas have a variety of existing applications, such as thin-film deposition, surface cleaning and sterilisation, and steel nitriding [1, 2]. Whilst many current applications are concerned with low pressure operating systems, there are emerging uses for nitrogen LTPs in the fields of biomedicine, agriculture and chemical processing, which benefit from operation at atmospheric pressure. The rationale behind the application of LTPs in these areas relies on making use of the rich cocktail of reactive nitrogen species, electric fields and charged particles they produce, to elicit biological responses in treated soil, seeds or biological tissues, or in using the non-equilibrium chemical kinetics for energy efficient chemical conversion. For example, there is potential for using nitrogen LTPs to increase the rate of seed germination, increase seedling growth, reduce seed contamination and aid nitrogen fixation in soil [3–6]. Similarly, the same rich cocktail of active components is under investigation for therapeutic purposes, such as for chronic wound and cancer treatments [7, 8].

In all applications, understanding the underlying chemical kinetics that control the production of important reactive species is vital, in order to develop optimal plasma compositions and treatments in each case. Of particular importance is the link between the chemical kinetics and experimental operating parameters, such as the applied power or the characteristics of the voltage waveform driving the plasma. This relationship needs to be understood to allow tailoring of specific plasma-produced species.

Nitrogen, as with other diatomic molecules, can exist in many electronically and vibrationally excited states. In nitrogen, the electron impact cross sections for the excitation of vibrational states from the ground state are large. This means that low lying vibrationally excited states are significantly populated by electron-impact excitation, and their production is a major energy loss mechanism for electrons [9]. On top of this, due to the high dissociation energy of the nitrogen molecule, vibrationally excited states of nitrogen can carry energies of up to ≈9 eV [10]. These high energies allow vibrationally excited nitrogen molecules to actively participate in the overall plasma chemistry by influencing the densities of molecular metastables that lie at similar energy levels. For example, molecular and atomic metastables of N_2_ (N$_2(A, B, a^{\prime})$) and N (N$(^2P, ^2D$)) all lie in a similar energy range (approx. 6–9 eV) as the upper vibrational levels [10]. It is known that at low pressures, the degree of vibrational excitation in nitrogen LTPs can have a strong influence on the chemical kinetics of other species, both in active discharges and associated afterglows [9, 11–14]. Therefore, it is important to determine whether vibrational excitation is similarly influential for the overall chemistry occurring in atmospheric pressure nitrogen LTPs.

Further, the energies of vibrationally excited nitrogen molecules can be equal to, or greater than, that of many organic bonds. Therefore, their potential for directly influencing bio-molecules and biological pathways should also be considered. For example, carbon–hydrogen (C–H) bonds in saturated hydrocarbons have a bond energy of ≈400 kJ/mole (≈4.1 eV) [15], which is exceeded by the energies of nitrogen vibrational levels of *v* = 15 and above. In addition, when oxygen is present, vibrationally excited nitrogen can play a significant role in the production of nitric oxide (NO). This is an important species in the fields of biomedicine, as NO can influence blood pressure regulation, immune responses and wound healing [16–18], and agriculture, where it can contribute to nitrogen fixation processes [19]. The production of NO can occur via the Zel’dovich reaction as follows [5, 20, 21]:

\begin{equation*} \mathrm {N}_2(v \geqslant 13) + \mathrm {O} \rightarrow \mathrm {NO + N}. \end{equation*}

Therefore, elucidation of the vibrational kinetics and densities of vibrationally excited states in atmospheric pressure nitrogen discharges should allow a deeper understanding of how the production of species, such as NO, can be promoted for specific applications.

The chemical kinetics in plasma sources produced in molecular nitrogen have been studied in detail in the literature under various conditions. Previous work concerning nitrogen plasmas can be broadly split into several groups. (a) Low pressure discharges, where discharge and afterglow chemical and vibrational kinetics have been interrogated by both experiment and modelling [9, 11, 13, 20–31]. These studies give insight into many of the basic chemical kinetics occurring in N_2_ plasmas. (b) High pressure nitrogen plasmas, where vibrational excitation has been investigated for plasmas produced by single pulses in modelling studies [32]. These studies are particularly interesting from a fundamental plasma physics perspective, but are not necessarily representative of the full range of effects that may occur in application-focused plasma sources that typically operate over longer timescales/multiple pulses. (c) High pressure, repetitively pulsed, or radiofrequency plasmas containing nitrogen (for example, using an air feed gas, or noble gases with a nitrogen admixture), where chemical kinetics are investigated, but without the inclusion of detailed vibrational kinetics [33–43]. (d) Studies over a variety of pressure ranges that have sought to identify the role of N_2_ vibrational excitation in chemical conversion applications, such as the production of NO or NH_3_ [44–46].

The research undertaken in low pressure nitrogen LTPs suggests the importance of vibrational excitation in mediating both powered and non-powered regions of a discharge. Since atmospheric pressure plasmas operating in molecular gases, such as nitrogen, are often powered by repetitive pulses, it is important to consider how vibrational states produced during one pulse period may influence subsequent pulses, by taking into account the afterglow between periods of power deposition. In this work, the chemical kinetics occurring in repetitively pulsed nitrogen discharges at atmospheric pressure are investigated through use of a 0-dimensional plasma chemical-kinetics model, GlobalKin [33]. Conditions typical of a dielectric barrier discharge (DBD) are modelled, with dimensions equivalent to the experimental system described in [47, 48]. The power input to the plasma is modelled as repetitive square-wave pulses, similar to the power inputs commonly used in experimental DBDs, and is suitable to describe the basic phenomena occurring in application-focused systems.

It is expected that a significant amount of the energy deposited into the discharge will lead to the excitation of vibrational levels under the conditions studied here. In general, the fraction of energy input into a plasma which is lost into different processes varies, depending on the reduced electric field (E/N) of the plasma. This has been discussed in the context of chemical conversion applications in a number of works, for example, [49]. At low values of E/N ($\lt$5 Td), energy is mainly deposited into rotational states, and at high values of E/N ($\gt$200 Td), energy is increasingly deposited into ionisation. Between these two extremes, significant energy is deposited into vibrational excitation. In this study, the peak E/N values at the time when power is applied are in the range of 80–120 Td, which falls in the range where strong vibrational excitation is expected. While this is well known, the longer-term kinetics of vibrational levels, after their initial formation by electron impact processes, has not been extensively studied at atmospheric pressure. This, is the key basis of the present work, where the effect of energy input on the excitation of vibrational states, in an atmospheric pressure plasma, is investigated. Specifically, the energy delivery is varied by changing the peak power and pulse frequency. These variation techniques are chosen as they are experimentally accessible, and can provide practical guidance to experimental works studying discharges operating within these commonly used parameter ranges.

## 2. Modelling and reaction scheme

### 2.1. GlobalKin

The plasma chemical-kinetics code, GlobalKin, described in [33, 50] is used to calculate the time-evolution of species densities and the electron temperature. Briefly, the model calculates the solution of coupled mass balance equations for each species, as shown in (2), taking into account species interactions at the surfaces (first term), and species formation and consumption due to the gas phase plasma-chemical reactions (second term)

\begin{equation*} \frac{\mathrm {d}n_i}{\mathrm {dt}} = \frac{S}{V}\Bigg(- \frac{D_in_i\gamma_i}{\gamma_i \Lambda_D + \frac{4 D_i}{\nu_{\mathrm {th},i}}} + \sum_j\frac{D_jn_j\gamma_jf_{ji}}{\gamma_j \Lambda_D + \frac{4 D_j}{\nu_{\mathrm {th},j}}} \Bigg) + S_i . \end{equation*}

Here, *n*_*i*_ is the number density of species *i*, *S*_*i*_ is the source term accounting for gas-phase plasma-chemical reactions and $\frac{S}{V}$ is the surface area to volume ratio of the plasma source. The surface interactions of different species are determined by certain properties: their sticking coefficients, *γ*, which is the probability of the species being lost to the surface; the diffusion coefficient, *D*, dependent on the properties of the species, and the surrounding gas; the diffusion distance, $\Lambda_D$; the return fraction, *f*_*ji*_, which is the fraction of species *j* lost, that returns to the gas-phase as species *i*; and the thermal velocity of species *i*, $\nu_{\mathrm {th}, i}$.

The electron temperature is determined by solving the electron energy conservation equation, where the external power deposition into the system, $P_\mathrm {d}$, acts as a source term. Electron energy losses are given by elastic and inelastic collisions, accounted for by the second and third terms on the right hand side of (3). Energy is assumed to be entirely deposited in electrons initially, due to the low mobility of ions [33]

\begin{align*} \frac{\mathrm {d}}{\mathrm {dt}}\Big(\frac{3}{2}n_\mathrm {e}k_\mathrm {B}T_\mathrm {e}\Big) &amp;= P_\mathrm {d} - \sum_i\frac{3}{2}n_\mathrm {e}\nu_{mi}\Big(\frac{2m_\mathrm {e}}{M_i}\Big)k_\mathrm {B}(T_\mathrm {e} - T_\mathrm {i})\nonumber\\ &amp;\quad + \sum_l n_\mathrm {e}k_\mathrm {l}n_\mathrm {l}\Delta\epsilon_\mathrm {l}. \end{align*}

Here, $n_\mathrm {e}$ is the electron number density; $T_\mathrm {e}$ and $T_\mathrm {i}$ are the electron and heavy particle temperatures, respectively; $m_\mathrm {e}$ and $M_\mathrm {i}$ are the masses of electrons and heavy particles, respectively. *ν*_*mi*_ is the electron collision frequency, *k* is the reaction rate coefficient and $\Delta \epsilon_\mathrm {l}$ is the electron energy change through inelastic collisions. $k_\mathrm {B}$ is the Boltzmann constant.

The electron energy distribution function (EEDF) is determined using the two-term approximation of the Boltzmann equation, using electron impact cross sections as input. The consistent set of electron impact cross sections used are described in the next section. The solver is called at regular, user-defined time intervals and provides electron mobilities and electron impact reaction rate coefficients for use in the mass conservation and electron energy conservation equations.

### 2.2. Plasma-chemical reaction scheme

A plasma-chemical reaction scheme for the simulation of atmospheric pressure nitrogen discharges has been developed, with special attention paid to the vibrationally excited states of the electronic ground state of nitrogen. The reaction scheme presented here is developed from the works presented in [9, 21, 24, 51], where reaction schemes for a range of pressures have been constructed and discussed in detail. Particular emphasis is placed on the vibrational kinetics, with electron impact excitation of vibrational levels from ground state nitrogen molecules included using the cross section data from [10]. As per [21, 52], vibrational state resolved vibration–vibration (V–V) and vibration–translation (V–T) reaction rate coefficients are calculated using a method based on the Schwartz, Slawsky and Herzfeld (SSH) theory [53–56], which is described in detail in appendices A and B.

The species included in the reaction scheme are shown in table 1. Alongside electrons, ground state molecular nitrogen (N$_2(X)$), electronically excited nitrogen (N$_2(A^3\Sigma_u^+, B^3\Pi_g, B^{^{\prime} 3}\Sigma_u^-, W^3\Delta_u, C^3\Pi_u, E^3\Sigma_g^+, a^1\Pi_g,$ $a^{^{\prime} 1}\Sigma_u^-, w^1\Delta_u)$, N$(^2P, ^2D)$) and nitrogen ions (N$_4^+$, N$_2^+(X, B)$ and N$^+(^3P)$) are included. Some of the electronically excited states of N_2_ are included as effective lumped states, rather than individual species. The radiative lifetimes of N$_2(a)$ and N$_2(w)$ are ≈$10^{-4}$ s, and both states undergo decay to produce the much longer lived N$_2(a^{\prime})$ metastable state with a lifetime of ≈0.5 s [57]. In addition, N$_2(a)$ and N$_2(w)$ are converted into N$_2(a^{\prime})$ through collisional quenching with N_2_ with a high reaction rate coefficient of ≈$10^{-11}$ cm^3^ s^−1^, meaning that the three states are strongly coupled [21, 24]. Therefore, only N$_2(a^{\prime})$ is included in the reaction scheme, and the cross section for electron impact excitation of N$_2(a^{\prime})$ is the sum of the cross sections for the individual states. Similarly, N$_2(B)$ and N$_2(W)$ are considered as a lumped state as per [9]. Finally, the N$_2(C)$ and N$_2(E)$ triplet states are also considered as a single state, as in [24]. For the starting conditions, N_2_ has an initial molar fraction of 1 (MF$_{\mathrm{N}_2}$ = 1), while the molar fraction of electrons is MF$_\mathrm{e} = 10^{-11}$. Ions and electronically excited states have MF values of $10^{-12}-10^{-10}$. The initial MF of vibrationally excited states are determined by a Boltzmann distribution at 310 K.

**Table 1.** Species included in the reaction scheme.

Particle	State
N_2_	$ X(v=0-45), A, (\textbf{B},W)^1, B^{^{\prime}}, (\textbf{C},E)$ ^a^, $(\textbf{a}^{^{\prime}}, a, w)$ ^a^
N	$^4S, ^2P, ^2D$
N$_4^+$	*X*
N$_2^+$	$ X, B $
N^+^	*X*
$e^-$	—

^a^ Species considered as a single lumped state. The name of the lumped state is denoted by the species in bold.

Reactions for each species at surfaces are specified for use in (2). For ground state molecular nitrogen, $\gamma_{\mathrm {N}_2} = 0$, meaning there is no loss at the walls. For positive ions and electronically excited species, $\gamma_+ = \gamma_* = 1$, and $f_+ = f_* = 1$ where the return species is the neutral, ground state of the species. Electrons are assumed to be lost to the surfaces with $\gamma_\mathrm {e} = 1$ and $f_\mathrm {e} = 0$.

Exceptions to these generalisations in this case are the vibrationally excited states of nitrogen, and atomic nitrogen. For N$_2(X, v)$, $\gamma_{\mathrm {vib}} = 4.5 \times 10^{-4}$ [58, 59] and $f_{\mathrm {vib}} = 1$, returning as N$_2(X, v-1)$ [21, 42]. For N, a value of $\gamma_\mathrm {N} = 1 \times 10^{-4}$ was chosen to be suitable, with all lost N returning as N_2_. While this value is an estimate, it falls within the assumed range of N wall losses in low pressure discharges of $3 \times 10^{-6}$ and $1 \times 10^{-3}$ [9].

Table 2 shows the consistent set of electron impact reactions included in the model, their threshold energies, the original reference for the cross section data, and any notes about their use in the reaction scheme. All reaction rate coefficients for the electron impact reactions are calculated as a function of the electron temperature from the EEDFs calculated by the solution of the two-term approximation of the Boltzmann equation.

**Table 2.** Electron impact reactions.

No.	$E_{\mathrm {Thr}}$ (eV)	Reaction	Rate	Reference	Note
Rotational excitation					
1	0.002	$e^- + \mathrm {N}_2 \rightarrow \mathrm {N}_2(r) + e^-$	*f*(*E*)	[61]	
Momentum transfer					
2	0.0	$e^- + \mathrm {N}_2 \rightarrow \mathrm {N}_2 + e^-$	*f*(*E*)	[61]	
3	0.0	$e^- + \mathrm {N}_2(A) \rightarrow \mathrm {N}_2(A) + e^-$	*f*(*E*)	[61]	^a^
4	0.0	$e^- + \mathrm {N}_2(a^{\prime}) \rightarrow \mathrm {N}_2(a^{\prime}) + e^-$	*f*(*E*)	[61]	^a^
5	0.0	$e^- + \mathrm {N} \rightarrow \mathrm {N} + e^-$	*f*(*E*)	[62]	
6	0.0	$e^- + \mathrm {N}^+ \rightarrow \mathrm {N}^+ + e^-$	*f*(*E*)	—	^b^
7	0.0	$e^- + \mathrm {N}_2^+ \rightarrow \mathrm {N}_2^+ + e^-$	*f*(*E*)	—	^b^
8	0.0	$e^- + \mathrm {N}_4^+ \rightarrow \mathrm {N}_4^+ + e^-$	*f*(*E*)	—	^b^
Excitation					
9	6.17	$e^- + \mathrm {N}_2(X) \rightleftharpoons \mathrm {N}_2(A) + e^-$	*f*(*E*)	[61]	^c^
10	8.40	$e^- + \mathrm {N}_2(X) \rightleftharpoons \mathrm {N}_2(a^{\prime},a,w) + e^-$	*f*(*E*)	[57, 63]	^c^,^d^
11	7.35	$e^- + \mathrm {N}_2(X) \rightleftharpoons \mathrm {N}_2(B,W) + e^-$	*f*(*E*)	[63, 64]	^c^,^d^
12	8.16	$e^- + \mathrm {N}_2(X) \rightleftharpoons \mathrm {N}_2(B^{^{\prime}}) + e^-$	*f*(*E*)	[63, 64]	^c^
13	11.1	$e^- + \mathrm {N}_2(X) \rightleftharpoons \mathrm {N}_2(C,E) + e^-$	*f*(*E*)	[61]	^c^,^e^
14	0.288–9.163	$e^- + \mathrm {N}_2(X,v=0) \rightleftharpoons \mathrm {N}_2(X,v\gt0) + e^-$	*f*(*E*)	[10]	^c^
15	2.38	$e^- + \mathrm {N}(^4S) \rightleftharpoons \mathrm {N}(^2D) + e^-$	*f*(*E*)	[65]	^c^
16	3.57	$e^- + \mathrm {N}(^4S) \rightleftharpoons \mathrm {N}(^2P) + e^-$	*f*(*E*)	[65]	^c^
Ionisation					
17	15.5	$e^- + \mathrm {N}_2 \rightarrow \mathrm {N}_2^+ + e^- + e^-$	*f*(*E*)	[61]	
18	9.33	$e^- + \mathrm {N}_2(A) \rightarrow \mathrm {N}_2^+ + e^- + e^-$	*f*(*E*)	[66]	
19	8.8	$e^- + \mathrm {N}_2(a^{\prime}) \rightarrow \mathrm {N}_2^+ + e^- + e^-$	*f*(*E*)	[67]	
20	14.55	$e^- + \mathrm {N} \rightarrow \mathrm {N}^+ + e^- + e^-$	*f*(*E*)	[68]	^e^
Dissociation					
21	9.76	$e^- + \mathrm {N}_2 \rightarrow \mathrm {N} + \mathrm {N} + e^-$	*f*(*E*)	[61]	
22	24.3	$e^- + \mathrm {N}_2 \rightarrow \mathrm {N}^+ + \mathrm {N} + e^- + e^-$	*f*(*E*)	[61]	
(Dissociative) Electron-ion recombination					
23	0.0001	$e^- + \mathrm {N}^+ \rightarrow \mathrm {N}$	*f*(*E*)	[69]	
24	0.001	$e^- + \mathrm {N}_2^+ \rightarrow \mathrm {N} + \mathrm {N}$	*f*(*E*)	[60]	
25	0.001	$e^- + \mathrm {N}_4^+ \rightarrow \mathrm {N}_2 + \mathrm {N}_2$	*f*(*E*)	[60]	^f^

^a^ Cross section assumed to be the same as for the ground state.

^b^ Calculated using the Coulomb Logarithm.

^c^ Cross section for reverse process determined by the principle of detailed balance.

^d^ Cross section for formation of the lumped state calculated as the sum of the cross sections for the individual states.

^e^ Normalised Gryzinski approximation used for the cross section below 25 eV.

^f^ Cross section for electron recombination with N$_2^+$ is used with absolute value scaled based on the difference in rate coefficients specified in [60].

Table 3 shows all of the heavy particle reactions taken into account in the reaction scheme. Here, the reactions, their rate coefficients in cm^3^ s^−1^, cm^6^ s^−1^ or s^−1^ (for 2-body reactions, 3-body reactions and radiative processes, respectively), original reference, and notes about their implementation are shown.

**Table 3.** Heavy particle reactions.

No.	Reaction	Rate (cm^3^s^−1^/cm^6^s^−1^/s^−1^)	Reference	Note
Ion chemistry				
26	$\mathrm {N}_2(A) + \mathrm {N}_2(a^{\prime}) \rightarrow \mathrm {N}_4^+ + e^-$	$k=1 \times 10^{-11}$	[25]	
27	$\mathrm {N}_2(a^{\prime}) + \mathrm {N}_2(a^{\prime}) \rightarrow \mathrm {N}_4^+ + e^-$	$k=5 \times 10^{-11}$	[25]	
28	$\mathrm {N}(^2D) + \mathrm {N}(^2P) \rightarrow \mathrm {N}_2^+ + e^-$	$k=1 \times 10^{-13}$	[74]	
29	$\mathrm {N}_2^+ + \mathrm {N}_2 + \mathrm {N}_2 \rightarrow \mathrm {N}_4^+ + \mathrm {N}_2$	$k=6.8 \times 10^{-29}$	[75]	
30	$\mathrm {N}_2(12 \leqslant v \leqslant 17) + \mathrm {N}_2^+ \rightarrow \mathrm {N}_2^+(B) + \mathrm {N}_2(v-12)$	$k=1 \times 10^{-11}$	[28]	
Quenching by $\mathrm {N}_2$ and $\mathrm {N}$				
31	$\mathrm {N}(^2D) + \mathrm {N}_2 \rightarrow \mathrm {N} + \mathrm {N}_2$	$k=1 \times 10^{-13} \mathrm {exp}(-510/T)$	[76]	
32	$\mathrm {N}(^2P) + \mathrm {N} \rightarrow \mathrm {N} + \mathrm {N}$	$k=6.2 \times 10^{-13}$	[77, 78]	
33	$\mathrm {N}(^2P) + \mathrm {N}_2 \rightarrow \mathrm {N} + \mathrm {N}_2$	$k=3 \times 10^{-17}$	[79, 80]	
34	$\mathrm {N}_2(A) + \mathrm {N}_2 \rightarrow \mathrm {N}_2 + \mathrm {N}_2$	$k=2 \times 10^{-18}$	[81]	
35	$\mathrm {N}_2(B) + \mathrm {N}_2 \rightarrow \mathrm {N}_2(A) + \mathrm {N}_2$	$k=2.85 \times 10^{-11}$	[25, 82]	
36	$\mathrm {N}_2(B) + \mathrm {N}_2 \rightarrow \mathrm {N}_2 + \mathrm {N}_2$	$k=1.5 \times 10^{-12}$	[25, 82]	
37	$\mathrm {N}_2(C) + \mathrm {N}_2 \rightarrow \mathrm {N}_2(a^{\prime}) + \mathrm {N}_2$	$k=1.32 \times 10^{-11}$	[83]	
38	$\mathrm {N}_2(a^{\prime}) + \mathrm {N}_2 \rightarrow \mathrm {N}_2(B) + \mathrm {N}_2$	$k=1.9 \times 10^{-13}$	[84]	^a^
Radiative decay				
39	$\mathrm {N}_2(B) \rightarrow \mathrm {N}_2(A) + hv$	$k=1.62 \times 10^{5}$ s^−1^	[85]	
40	$\mathrm {N}_2(C) \rightarrow \mathrm {N}_2(B) + hv$	$k=2.74 \times 10^{7}$ s^−1^	[85]	
41	$\mathrm {N}_2(B^{^{\prime}}) \rightarrow \mathrm {N}_2(X) + hv$	$k=2.56 \times 10^4$	[85]	
42	$\mathrm {N}_2^+(B) \rightarrow \mathrm {N}_2^+(X) + hv$	$k=1.6 \times 10^{7}$ s^−1^	[85]	
Dissociation				
43	$2 \mathrm {N}_2(14 \leqslant v \leqslant 25) \rightarrow \mathrm {N}_2 + \mathrm {N} + \mathrm {N}$	$k=3.5 \times 10^{-15}$	[11]	^b^
44	$\mathrm {N}_2(A) + \mathrm {N}_2(14\leqslant v\leqslant19) \rightarrow \mathrm {N}_2 + \mathrm {N} + \mathrm {N}$	$k=1.8 \times 10^{-11} \mathrm {exp}(-1765/T)$	[11, 24]	
45	$\mathrm {N}_2(43 \leqslant v \leqslant 45) + \mathrm {N}_2 \rightarrow \mathrm {N}_2 + \mathrm {N} + \mathrm {N}$	$k=8.4 \times 10^{-17}$	—	^c^
Energy Pooling				
46	$\mathrm {N}_2(A) + \mathrm {N}_2(A) \rightarrow \mathrm {N}_2(B) + \mathrm {N}_2(v=8)$	$k=7.7 \times 10^{-11}$	[86]	
47	$\mathrm {N}_2(A) + \mathrm {N}_2(A) \rightarrow \mathrm {N}_2(C) + \mathrm {N}_2(v=2)$	$k=1.5 \times 10^{-10}$	[87]	
48	$\mathrm {N}_2(A) + \mathrm {N}_2(5\lt v\lt14) \rightarrow \mathrm {N}_2(B) + \mathrm {N}_2$	$k=2 \times 10^{-11}$	[25]	
49	$\mathrm {N}_2(A) + \mathrm {N} \rightarrow \mathrm {N}_2(v=8,9) + \mathrm {N}(^2P)$	$k=1.5 \times 10^{-11}$	[24, 78]	
50	$\mathrm {N}_2(A) + \mathrm {N} \rightarrow \mathrm {N}_2(7 \leqslant v \leqslant 13) + \mathrm {N}(^2D)$	$k=2.5 \times 10^{-11}$	[24, 78]	
51	$\mathrm {N} + \mathrm {N}_2(40 \leqslant v \leqslant 45) \rightarrow \mathrm {N}(^2D) + \mathrm {N}_2(A)$	$k= 10^{-11} - 10^{-14}$	[13]	^d^
52	$\mathrm {N} + \mathrm {N}_2(39 \leqslant v \leqslant 45) \rightarrow \mathrm {N} + \mathrm {N}_2(a^{\prime})$	$k= 10^{-11} - 10^{-14}$	[13]	^d^
3-body recombination				
53	$\mathrm {N} + \mathrm {N} + \mathrm {N}_2 \rightarrow \mathrm {N}_2 + \mathrm {N}_2$	$k=8.3 \times 10^{-34} \mathrm {exp}(500/T)$	[72, 73]	^e^
54	$\mathrm {N} + \mathrm {N} + \mathrm {N}_2 \rightarrow \mathrm {N}_2(A) + \mathrm {N}_2$	$k=1.7 \times 10^{-33}$	[72, 73]	^e^
55	$\mathrm {N} + \mathrm {N} + \mathrm {N} \rightarrow \mathrm {N}_2(A) + \mathrm {N}$	$k=1.4 \times 10^{-32}$	[72, 73]	^e^
56	$\mathrm {N} + \mathrm {N} + \mathrm {N}_2 \rightarrow \mathrm {N}_2(B) + \mathrm {N}_2$	$k=2.4 \times 10^{-33}$	[72, 73]	^e^
57	$\mathrm {N} + \mathrm {N} + \mathrm {N} \rightarrow \mathrm {N}_2(B) + \mathrm {N}$	$k=1.4 \times 10^{-32}$	[72, 73]	^e^
Vibrational Kinetics				
58	$\mathrm {N}_2(v) + \mathrm {N}_2(w-1) \rightleftharpoons \mathrm {N}_2(v-1) + \mathrm {N}_2(w)$	See appendix A	[21]	^f^
59	$\mathrm {N}_2(v) + \mathrm {N}_2 \rightleftharpoons \mathrm {N}_2(v-1) + \mathrm {N}_2$	See appendix B	[52]	^f^
60	$\mathrm {N}_2(v) + \mathrm {N} \rightleftharpoons \mathrm {N}_2(v^{^{\prime}}) + \mathrm {N} \quad \textrm{where}\quad v^{^{\prime}}-v\leqslant 5$	See appendix C	[9, 21]	^f^

^a^ N$_2(a^{\prime})$ data assumed.

^b^ Only exothermic reactions are included.

^c^ Rate coefficient estimated from the reverse V–Tm process for *v* = 45.

^d^ Rate coefficient varied in this study, see text for more details.

^e^ The total reaction rate coefficient for reactions 53–57 in an atmospheric pressure plasma was measured in [72] and found to have a value of $7.77 \pm 1.04 \times 10^{-33}$ cm^6^ s^−1^. This reaction rate coefficient can then be split between the constituent reactions, as found in [73], where N recombines to produce N_2_, N$_2(A)$ and N$_2(B)$.

^f^ Rate coefficients calculated at 300 K.

Heavy particle collisions between vibrationally excited states (V–V and V–T reactions) have also been included. This is due to their known importance in low pressure systems for influencing the nitrogen vibrational distribution function (VDF), which in turn, influences the EEDF, and overall plasma chemistry [9]. The role of these reactions will be discussed in more detail later. The expressions for calculating the reaction rate coefficients for each of these processes, involving each of the vibrationally excited states, are shown in the appendices, and are based on the works of [21, 52, 70]. For V–V reactions (reactions 58) and V–T processes between molecules (V–Tm, reactions 59), only single quantum transitions are included. For V–T processes involving atoms (V–Ta, reactions 60), multi-quantum transitions of up to five vibrational levels are also significant, therefore, they are included, with the reaction rate coefficient being kept constant for all transitions where $v^{^{\prime}} - v \leqslant 5$ [9, 52, 71].

With increasing pressure, 3-body recombination processes for N atoms are thought to become more important, therefore, these reactions are included as per the works of [72, 73].

### 2.3. Geometry, power input and gas temperature

With the exception of section 3.1, the system modelled in this work is a repetitively pulsed plasma source, with characteristics similar to a volume DBD, such as that studied in [47, 48]. Simulations are performed with the diffusion length and plasma volume specified for a cylindrical plasma-forming region of 1 cm diameter, with a 1 mm discharge gap. These are typical dimensions for DBD devices.

**Figure 1.**
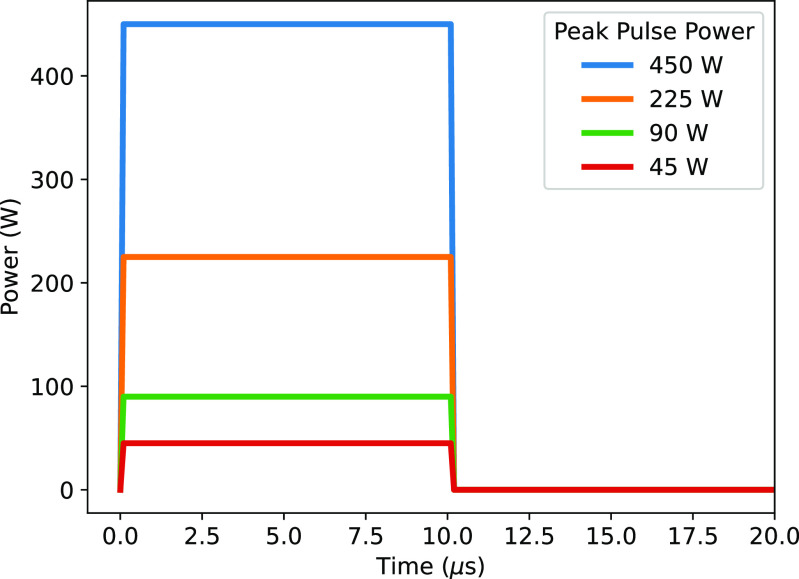
Pulse characteristics used in this modelling study, for a variety of peak pulse powers. The rise and fall times are 100 ns each, and the pulse width is 10 *µ*s. The full off-time is not shown on this figure, and will vary with frequency.

In this work, the energy is delivered to the plasma by means of square pulses, with a rise and fall time each of 100 ns, and a pulse width of 10 *µ*s in all cases. Using this shape, the peak power and the pulse frequency can be varied to determine the effects of altering the average power/energy input on reactive species densities. The pulse repetition frequency is varied from 0.5–10 kHz, and the peak pulse power is varied from 23–450 W. The shape of the pulse is shown in figure 1 for a variety of pulse powers (the full off-time between pulses is not shown).

In [47], a DBD with the same geometry as that modelled here is powered by damped sine wave voltage pulses, which deliver approximately 0.45 mJ of energy in the first two sine waves of the pulse (the only waves where current peaks appear). Therefore, this energy was taken to be the base case for this study. The base case is, therefore, a pulse of 45 W peak power, at 1 kHz frequency. By changing the pulse power or the frequency between $\frac{1}{2}\times$ and 10 × the base case, the energy delivered over a period of 1 ms is varied between 0.23 mJ and 4.5 mJ. This equates to an average plasma power of 0.23–4.5 W.

At this point, it is worth noting that DBDs operated in nitrogen, such as those considered in [47, 48], are typically filamentary in nature, and are not necessarily, quantitatively, well represented in 0-dimensional models. Therefore, to make detailed comparisons with a specific experimental system it is generally necessary to carefully match the model to the system using different adaptations of the basic global model framework, as has been done for DBD systems in [88–90], for example. In this work, the aim is not necessarily to provide a quantitative description of the kinetics occurring in these filamentary systems. Rather, the focus is to demonstrate the basic scaling of the longer-term chemical kinetics, in particular, those related to the vibrational levels, with the total energy deposited into the system, independently of the temporal or spatial distribution of this energy deposition.

Throughout this work, a fixed gas temperature of 310 K is used for all simulations. This can also be viewed as a limitation of the current approach. As the power input or frequency is increased, it is expected that the gas temperature would rise in experimental systems. This would lead to changes in reaction rate coefficients for V–V, V–T and other heavy particle reactions. The inclusion of variable or calculated gas temperatures for higher energy inputs, would be likely to quantitatively affect some of the results presented in this work, such as the timing of switching behaviour discussed in the next sections. This should be kept in mind for future experimental comparisons.

### 2.4. Pathways analysis

To assess the contribution of different reaction pathways to species production and consumption, the absolute reaction rates of the different reactions can be compared. The absolute reaction rate, *R*_*j*_ is defined as:

\begin{equation*} R_j(t) = n_{ja} \times n_{jb} \times k_j.\end{equation*}

Where $R_{j}(t)$ is the absolute rate of reaction *j* at time *t*, *n*_*ja*_ and *n*_*jb*_ are the densities of the reactants in reaction *j* (assuming a 2-body reaction), and *k*_*j*_ is the reaction rate coefficient for reaction *j*.

For this work, unless otherwise stated in the text, reactions with an absolute rate less than $1 \times 10^{10}$ cm^−3^ s^−1^ are ignored in the analysis.

## 3. Results

### 3.1. Experimental validation

As an initial validation of the reaction scheme developed in this work, and its application to repetitively pulsed plasma systems, simulations have been carried out for the conditions studied by Jans *et al* [91]. Here, the authors measured the densities of N$_2(A)$ in a flowing DBD operated in nitrogen at a pressure of 250 Torr. For the power input in the simulations, the ‘Simulation Power’ profile given in figure 2(a) of [91] was used. This peak power deposition is much higher (MW), and the duration of the power deposition is much shorter (ns), than that considered in the following sections of this work. Despite these differences, the choice to compare with the results of [91] was motivated by the high quality and time resolution of the measurements, and the lack of such measurements under the conditions studied in the following sections.

**Figure 2.**
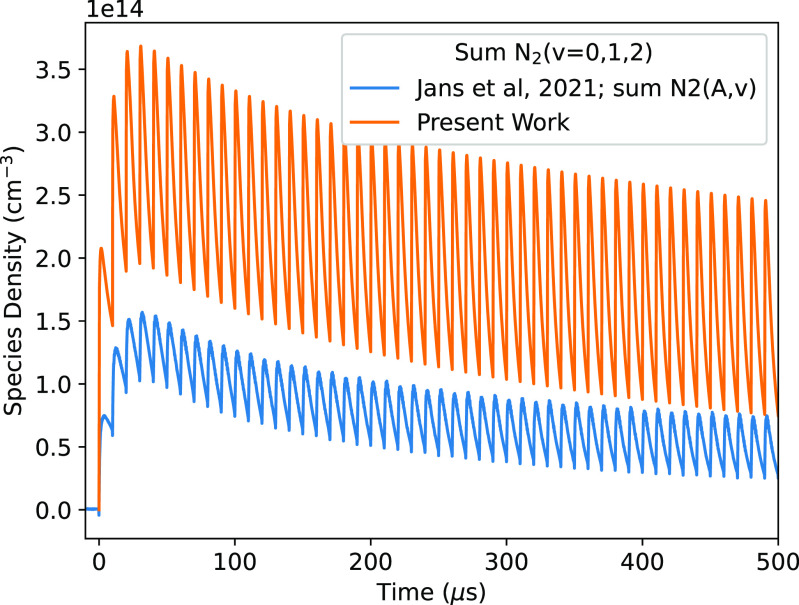
Comparison between measured and simulated N$_2(A)$ densities in a flowing pure nitrogen discharge at 250 Torr. Measurements have been reproduced with kind permission of the authors of [91].

Figure 2 shows the comparison between the published concentrations of N$_2(A)$, and those simulated using the reaction scheme developed in this work. In [91], the densities of N$_2(A, v = 0, 1, 2, 4, 5$) are all measured separately. Since these states are not included individually in our reaction scheme, we have added together the densities of each vibrational level measured in [91] and compare this summed density to the effective N$_2(A)$ from the simulations of this work. Here, it can be seen that the simulated densities of N$_2(A)$ are approximately a factor of 2 higher than the measured densities. The relative variation in the densities, from maximum to minimum, is similar when comparing experiment and simulation. Overall, this can be viewed as a reasonable agreement, in particular given the lack of a detailed description of the vibrational kinetics of N$_2(A)$ in our reaction scheme. It is also notable that the model used in [91] yields better quantitative agreement with the measured N$_2(A)$ densities. Though the reasons for this discrepancy have not been studied in detail, differences in the reaction scheme are likely to be an important factor.

### 3.2. Simulation base case

**Figure 3.**
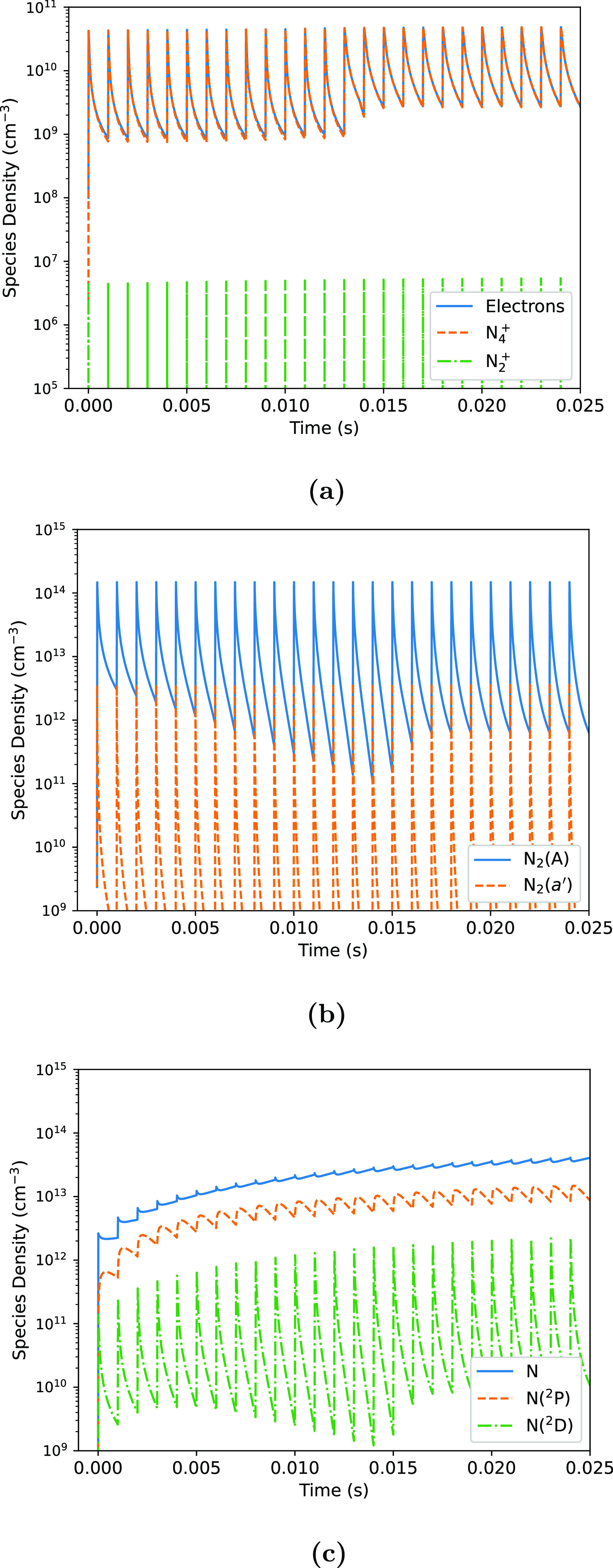
Densities of charged species (a), molecular metastables (b), and atomic species (c) in the base case simulation. The base case is defined as having a pulse repetition frequency of 1 kHz, a peak pulse power of 45 W, and a pulse width of 10 *µ*s.

In line with conditions typical of a DBD, the base case for this study is a square-wave pulse of 45 W peak power, repeated at 1 kHz frequency, delivering 0.45 mJ of energy in each pulse cycle (1 ms). The gas temperature is set at 310 K in all simulations. Figure 3 shows the time-evolution of the densities of a number of charged and neutral species under the base case conditions. Aside from atomic nitrogen, it can be seen that the densities of all species increase rapidly during the power on-time. They then decay rapidly during the power off-time due to an excess of recombination or quenching reactions occurring during the off-time. The maximum densities and decay rates vary between the species, but the trends remain consistent. In contrast, atomic nitrogen densities increase during the power on-time, but remain relatively constant during the off-time between pulses.

The electron density in this base case fluctuates with the pulse. Once the simulation has reached equilibrium (after ≈15 ms), the average electron density over a whole pulse period is ≈$1 \times 10^{10}$ cm^−3^. The equivalent experimental source investigated in [47] gives an average electron density of ≈$3 \times 10^9$ cm^−3^ (this work is approximately equivalent to the 18 kV pure nitrogen case in [47]). The factor of ≈3 between these two values can be considered a reasonable agreement, given the difference in pulse shapes, and intrinsic errors in measuring plasma power in the experimental setup, which is an input to the simulation.

During the power on-time, electrons and N$_2^+$ are formed mainly by electron impact ionisation (reaction 17). Due to the high pressure conditions, N$_2^+$ is rapidly converted into N$_4^+$ via the three body ion-molecule association reaction:

\begin{equation*} \textbf{rxn 29:} \quad \mathrm {N_2^+ + N_2 + N_2 \rightarrow N_4^+ + N_2}. \end{equation*}

This reaction serves to keep the density of N$_2^+$ low at all times, and the dominant ion is N$_4^+$. During the power off-time following each pulse, the electron-ion recombination reactions between electrons and N$_2^+$/N$_4^+$ (reactions 24/25) lead to a decrease in the densities of all three species.

While other species decrease during the power off-time, for ground state N atoms, production occurs in both the power on- and power off-times. The increase in N density during the pulse is mainly due to electron impact dissociation of N_2_ (reaction 21). However, during the off-time, destruction processes of electronically excited atomic nitrogen (N$(^2P)$ and N$(^2D)$) atoms act as a production mechanism for ground state N. Of particular importance is the quenching of N$(^2P)$ and N$(^2D)$ by N_2_, which results in the production of N and N_2_ (reactions 31 and 33). This reaction has a consistently high absolute rate during the power off-time, and allows the N density to remain high, and even increase, due to the long lifetime of N$(^2P)$ seen in figure 3. The main destruction mechanisms for N are:

\begin{equation*} \textbf{rxn 49:} \quad \mathrm {N}_2(A) + \mathrm {N} \rightarrow \mathrm {N}_2(v=8,9) + \mathrm {N}(^2P) \end{equation*}

\begin{equation*} \textbf{rxn 50:} \quad \mathrm {N}_2(A) + \mathrm {N \rightarrow N}_2(7 \leqslant v \leqslant 13) + \mathrm {N}(^2D). \end{equation*}

These serve to produce N$(^2D)$ and N$(^2P)$, which can then be recycled to produce N. Due to the low fractional density of N atoms, 3-body recombination reactions (reactions 53–57) have a consistently low absolute rate, and have only a small contribution to the destruction of N. Since the absolute rate of the N consuming reactions is consistently low, the result is a long lifetime for N, something which has also been observed experimentally by Es-Sebbar *et al* [92].

### 3.3. Power variation

By changing the peak pulse power, the energy input to the plasma can be varied. Here, a peak pulse power variation was performed to determine the effects on the kinetics and densities of different species. For this, species are split into vibrationally excited states, and non-vibrationally excited species, which are considered separately initially. The broader effects of the vibrationally excited states are then investigated and discussed.

#### 3.3.1. Vibrationally excited states.

**Figure 4.**
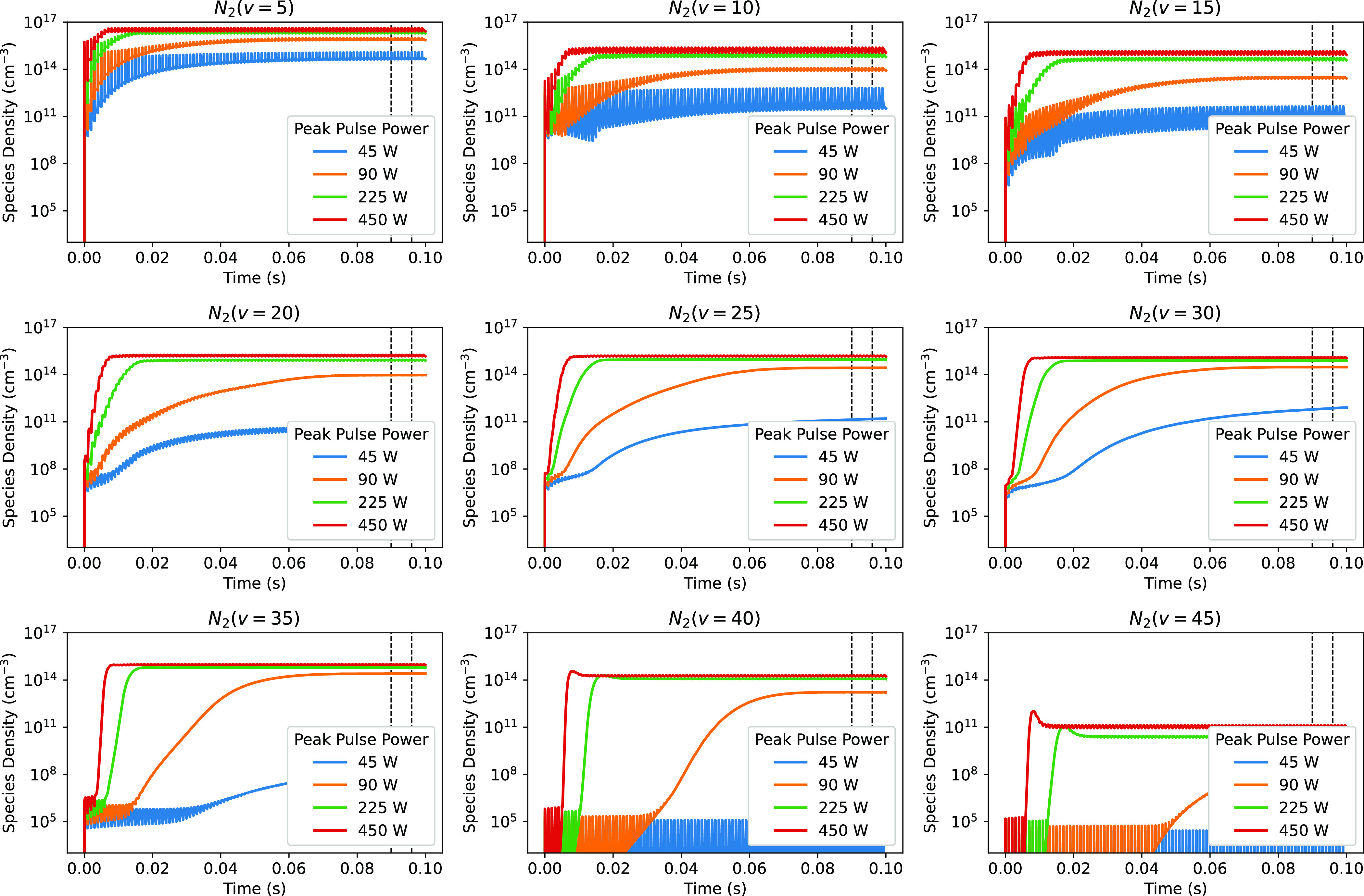
The time evolution of the densities of N$_2(v = 5,10,15,20,25,30,35,40,45)$ as a function of peak pulse power. For each panel, the base case (45 W) is the blue line showing the lowest densities, with progressively higher densities observed at 90 W, 225 W and 450 W, respectively. For all cases, the pulse repetition frequency remains at 1 kHz and the pulse width at 10 *µ*s. The vertical dashed black lines at 90 ms and 96 ms signify the time period for obtaining the average species densities shown in figures 5, 7 and 9.

Figure 4 shows the time evolution of the densities of N$_2(v = 5,10,15,20,25,30,35,40,45)$ over the full simulation time of 0.1 s (sufficient for densities to reach a periodic steady state). For all of the species shown up to, and including N$_2(v = 35)$, the densities increase over time, with higher equilibrium densities being achieved at higher peak powers. However, for the highest vibrationally excited states shown (N$_2(v = 40, 45)$), only peak pulse powers greater than 45 W result in a strong increase in density between the start and end of the simulation time, meaning that these states are only significantly populated in higher peak power cases.

However, while this general trend is observed, there are more subtle differences between the kinetics of the different vibrationally excited states as well. The species shown in figure 4 can be split into three groups of low, medium and high level vibrationally excited states, and for each of these groups, different kinetics are observed. For low level vibrational states ($v \lt \approx20$, top row), the densities increase throughout the simulation time, until they reach a power-dependent pulsed steady-state density. However, the densities throughout the simulation are strongly influenced by the power input occurring every 1 ms. This is shown by the large fluctuations in their densities and is particularly noticeable for the lower power cases (≈45–90 W).

Moving to the medium level vibrationally excited states (≈$20 \leqslant v \leqslant 30$, middle row), the kinetics start to change. For these states, initially, densities show small fluctuations that coincide with the power pulses. However, as time progresses, the densities start to increase significantly in a smooth and consistent manner. The densities still fluctuate in time with the power pulse, however, the extent of the fluctuation relative to the much larger density means that it is masked by the log scale, and is less important than at earlier time points. The rate of these increases (the gradients of the lines) are power dependent, with high powers showing faster density increases. For each of the power cases greater than 90 W, the densities reach a plateau value.

For the highest level vibrationally excited states (≈$v \gt35$), the kinetics are split between the low power cases (45 W) and the higher power cases ($\geqslant90$ W). For the low power cases, the kinetics are similar to those seen in the low level vibrationally excited states—the densities fluctuate with the power pulses. However, unlike the low level states, the densities of these high level vibrationally excited states in low power cases remain very low throughout the simulation time (maximum density $\ll1 \times 10^{7}$ cm^−3^). When comparing the trends of the high level vibrationally excited state densities with increasing power, there is a distinctive switch in kinetics for peak powers $\geqslant90$ W. Initially these species show fluctuations in density, remaining at similar densities to the lower powers. However, at a certain time point, their kinetics change significantly, with a rapid increase in density occurring, followed by an equilibrium density that is many orders of magnitude greater than the starting density. From the point that the density starts to increase rapidly, the dependence on the power input during the pulse on-time is reduced. This equilibrium density differs for each vibrationally excited state, but is largely consistent for all power cases above 90 W, particularly for N$_2(v = 35)$ and N$_2(v = 40)$. This suggests that the species densities approach a saturation point, and that increasing the power further has a less significant effect on their densities. This gives an interesting insight into the way of potentially producing vibrationally excited states in the most energy efficient way. For example, figure 4 shows that for the 225 W and 450 W peak pulse power cases, a plateau density for $v = 20-40$ of ≈$10^{15}$ cm^−3^ is reached just 20 ms after the start of the simulation. Therefore, there may be opportunities to optimise the energy efficiency of vibrationally excited state production by alternating periods of pulsing with periods where no power is applied. Ideally, the pulsed time would be long enough that the plateau could be reached (here ≈20 ms), and the peak pulse power would need to be sufficient for the maximum plateau to be reached.

#### 3.3.2. Vibrationally excited state production mechanisms.

The switch from pulse-dependence to pulse-independence seen in the high vibrational states suggests that there is a change in how these species are being produced at a certain time point in the simulation. Further to this, the mechanism that allows the switch in kinetics to occur is power dependent, and does not happen in the lowest power cases.

**Figure 5.**
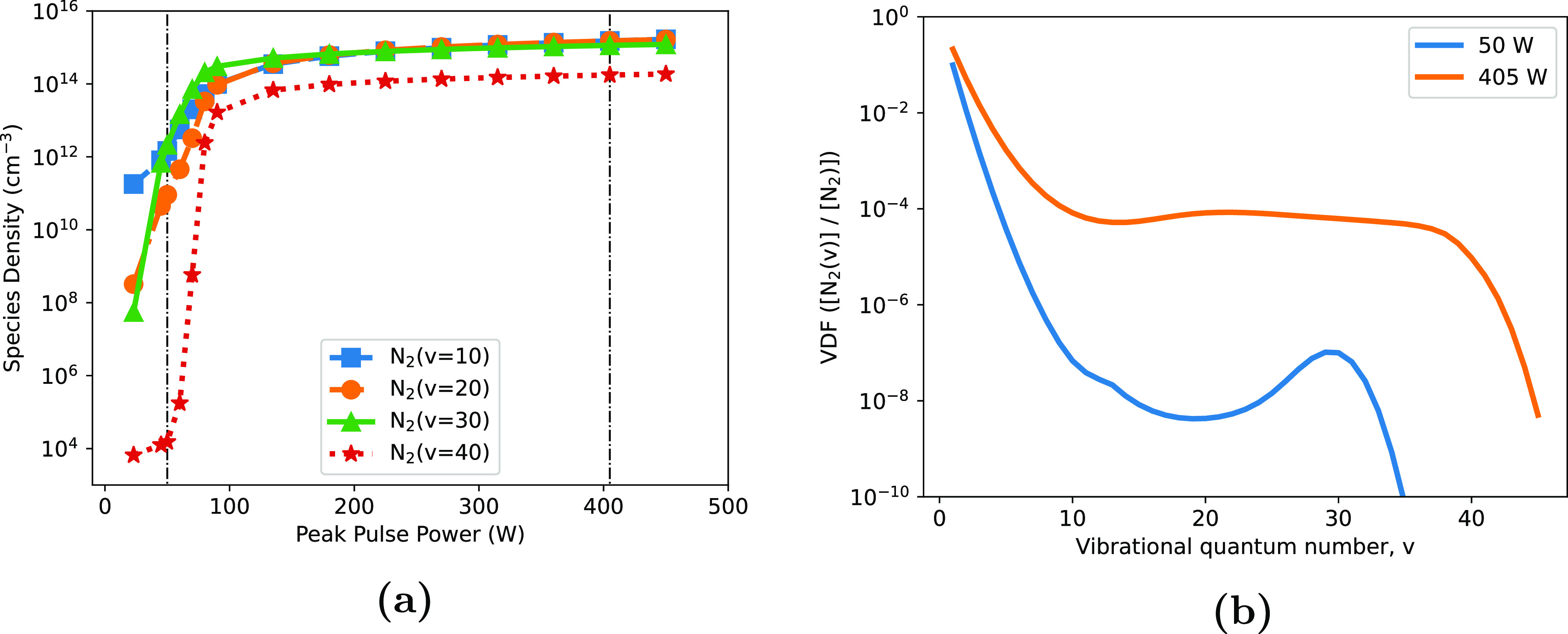
Average densities of vibrationally excited states. (a) Shows the equilibrium densities of N$_2(v = 10,20,30,40)$ as a function of peak pulse power. The densities were calculated by averaging over the time period 90–96 ms, as shown by the vertical dashed black lines in figure 4. (b) Shows the average VDFs over the same 90–96 ms period, in the 50 W and 405 W cases (corresponding to the vertical dash-dot lines in (a)). The frequency and pulse width were constant at 1 kHz and 10 *µ*s, respectively.

For each of the vibrationally excited states shown in figure 4, there appears to be a plateau density that is reached by the 225 W and 450 W cases, and in some cases the 90 W case. However, for the 45 W base case, this plateau is never reached, and the densities in this low power case are often orders of magnitude lower than the densities in the higher power cases. This is shown in more detail in figure 5(a), where the average equilibrium density of N$_2(v = 10,20,30,40)$, over the time region indicated by the vertical dashed black lines in figure 4, is shown as a function of peak pulse power. There is a striking increase in density between the 45 W and 90 W power cases, of ≈3–10 orders of magnitude, for N$_2(v = 10)$ and N$_2(v = 40)$, respectively. However, above this peak pulse power range, there is no further increase in species density, thereby reiterating the notion of a plateau density of vibrational states. In figure 5(b), average VDFs (averaged between 90–96 ms) are plotted for the 50 W and 405 W peak pulse power cases (corresponding to the vertical dash-dot lines in figure 5(a)). Here, the high power VDF appears to be significantly populated, right up to ≈$v = 40$, and there is a plateau region at ≈$10 \lt v \lt 37$. A similar plateau feature is also seen in [93]. However, in the low power case, the VDF is far less populated, and no plateau region exists.

To explain the change in behaviour between the low, medium and high level vibrationally excited states shown in figure 4, and the vast increase in vibrationally excited state density between 45 W and 90 W shown in figure 5, the production mechanisms of the vibrationally excited states are considered.

The densities of vibrationally excited states are largely influenced by two groups of reactions:

(a) Electron impact processes (elastic and superelastic)
  \begin{equation*} \textbf{rxn 14:} \quad e^- + \mathrm {N}_2(v=0) \rightleftharpoons \mathrm {N}_2(v\gt0) + e^- \end{equation*}
(b) Vibrational energy transfer processes:
  • Vibration-vibration (V–V) reactions:
    \begin{equation*} \textbf{rxn 58:} \quad \mathrm {N}_2(v) + \mathrm {N}_2(w-1) \rightleftharpoons \mathrm {N}_2(v-1) + \mathrm {N}_2(w) \end{equation*}
  • Molecular vibration–translation (V–Tm) reactions:
    \begin{equation*} \textbf{rxn 59:} \quad \mathrm {N}_2(v) + \mathrm {N}_2 \rightleftharpoons \mathrm {N}_2(v-1) + \mathrm {N}_2 \end{equation*}
  • Atomic vibration–translation (V–Ta) reactions:
    \begin{equation*} \textbf{rxn 60:} \quad \mathrm {N}_2(v) + \mathrm {N} \rightleftharpoons \mathrm {N}_2(v^{^{\prime}}) + \mathrm {N} \quad \textrm{where} \quad v-v^{^{\prime}} \leqslant 5. \end{equation*}

**Figure 6.**
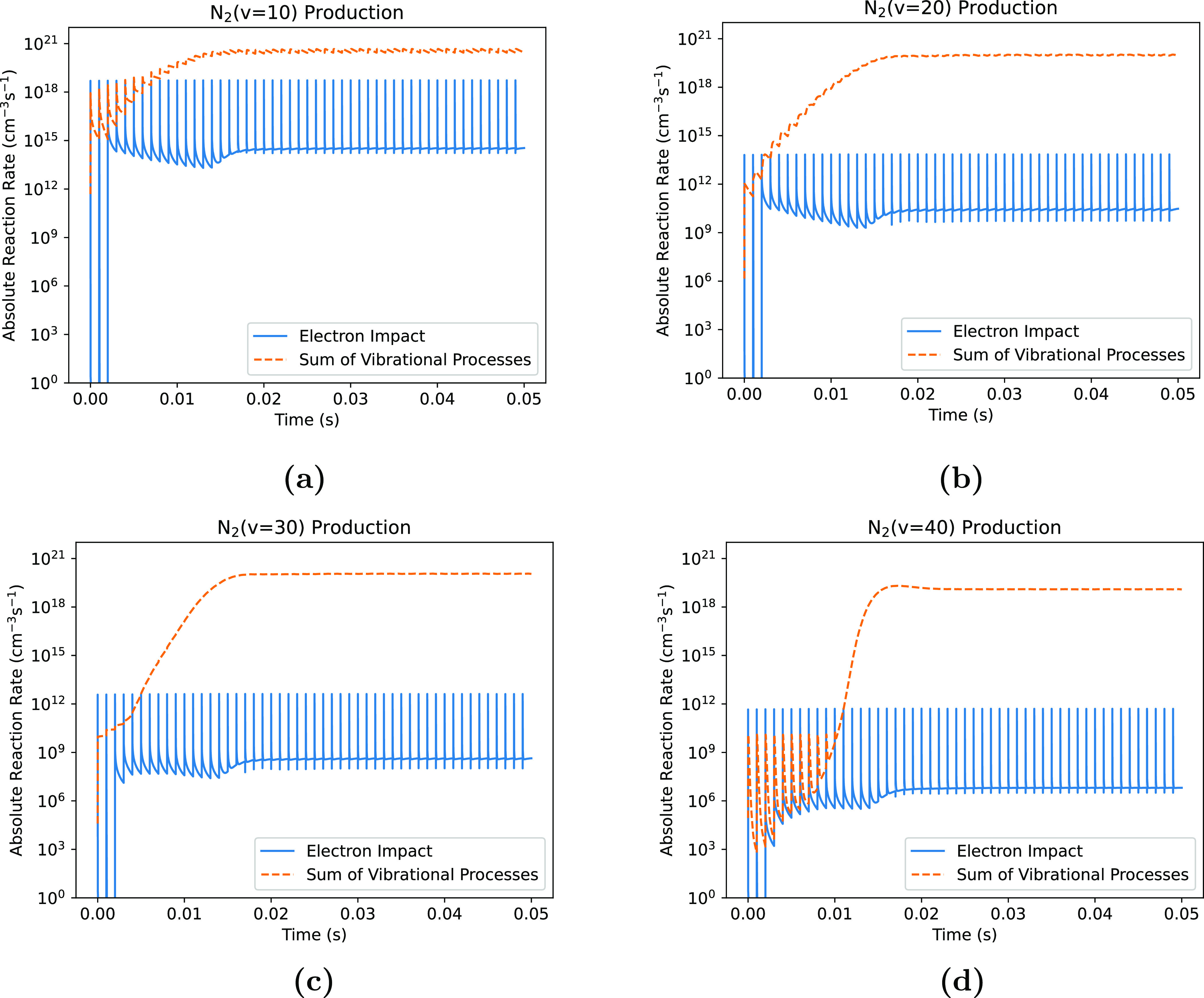
Comparison of the production of N$_2(v = 10)$ (a), N$_2(v = 20)$ (b), N$_2(v = 30)$ (c), and N$_2(v = 40)$ (d) via electron impact processes and vibrational energy transfer processes. ‘Sum of Vibrational Processes’ represents the total reaction rate for all V–V, V–Ta and V–Tm processes which result in the formation of the state in question. Data is from the 225 W peak pulse power case, with a pulse repetition frequency of 1 kHz and a pulse width of 10 *µ*s.

To compare the influence of these different mechanisms over time, the production of different vibrationally excited states through electron impact and vibrational energy transfer processes are shown in figure 6, using a peak power of 225 W as a representative case. In figure 6, the solid blue line shows the absolute electron impact excitation rates for each of N$_2(v = 10,20,30,40)$ as a function of time throughout the simulation. Generally, these reactions show a regularly fluctuating rate, with a peak occurring during the power on-time when the electron density is high, followed by a fast decay when the electron density decreases.

The orange dashed line in figure 6 shows the sum of all vibrational energy transfer processes (V–V, V–Tm and V–Ta) contributing to the production of that particular vibrationally excited state. In contrast to the electron impact excitation reactions, these processes have a very different temporal profile. The sum of the absolute reaction rates for these vibrational energy transfer processes initially fluctuates coherently with the power input. However, at a certain time point, the rates for these processes increase smoothly over time, and lose their direct dependence on individual power pulses. At this point, the vibrational energy transfer reaction rates rise rapidly and overtake the maximum electron impact excitation rates during the power on-time of the pulse. When the vibrational energy transfer reactions overtake the electron impact reactions, the production of vibrationally excited states can be described as changing from being electron-mediated to being vibrational energy transfer-mediated. It is important to note, that the same conclusion is reached when a similar analysis is performed where the **net** vibrational state production is compared to electron impact production (rather than only the vibrational production mechanisms, data not shown).

As shown in figure 6, the time point for the switch is dependent on the specific vibrationally excited state. It is also dependent on the value of the peak power, with higher power cases switching earlier than lower ones (data not shown).

#### 3.3.3. Non-vibrationally excited species.

**Figure 7.**
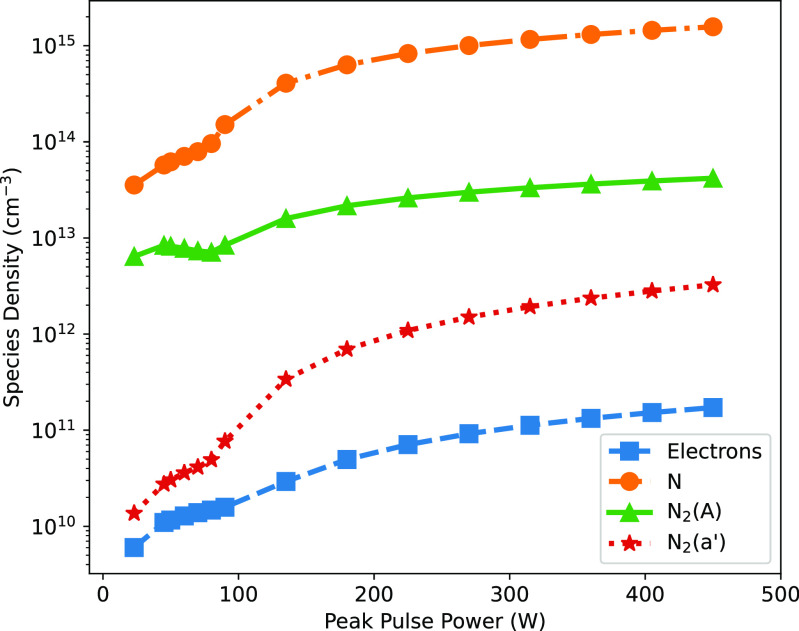
Average densities of electrons, N, N$_2(A)$ and N$_2(a^{\prime})$ during the equilibrium phase of the simulation, as a function of peak pulse power. The average density is calculated over the period 90–96 ms denoted by the vertical black lines in figure 4. The frequency and pulse width were constant at 1 kHz and 10 *µ*s, respectively.

Varying the peak pulse power also affects other species in the plasma, besides the vibrationally excited states. Figure 7, shows the average densities of electrons, N, N$_2(A)$, and N$_2(a^{\prime})$, between 90–96 ms, as a function of the peak pulse power. This figure shows that the average equilibrium densities of each of these species rises with increasing peak power. However, in comparison to figure 5 where vibrationally excited states were discussed, non-vibrationally excited species do not show a large jump in density between the 45 W and 90 W peak power cases.

The mechanisms by which the increased peak power results in an increased average density of N, electrons and metastable states will be discussed in later sections.

From experimental data available in the literature, the overall densities of N seen in this present study are reasonable. As mentioned above in section 3.2, experimental data available in [92] suggest that the maximum density for N in the energy deposition range of the present simulations is $3 \times 10^{14}$ cm^−3^. This is broadly consistent with the range of densities predicted by the simulations in this work. However, given the differences between the experimental setup used in that work, and that simulated here, we do not attempt a rigorous comparison. The order of magnitude of the presented N densities also agrees with other atmospheric pressure nitrogen discharge N densities, such as those presented in [94].

### 3.4. Pulse repetition frequency variation

A common mechanism of controlling the energy input to pulsed plasmas is to vary the pulse repetition frequency. Similar to the investigation into the effects of varying the peak pulse power, here, a pulse repetition frequency variation has been performed to determine the effects on vibrationally excited states, electrons, atomic nitrogen and the longer-lived metastable states, N$_2(A)$ and N$_2(a^{\prime})$.

#### 3.4.1. Vibrationally excited states.

**Figure 8.**
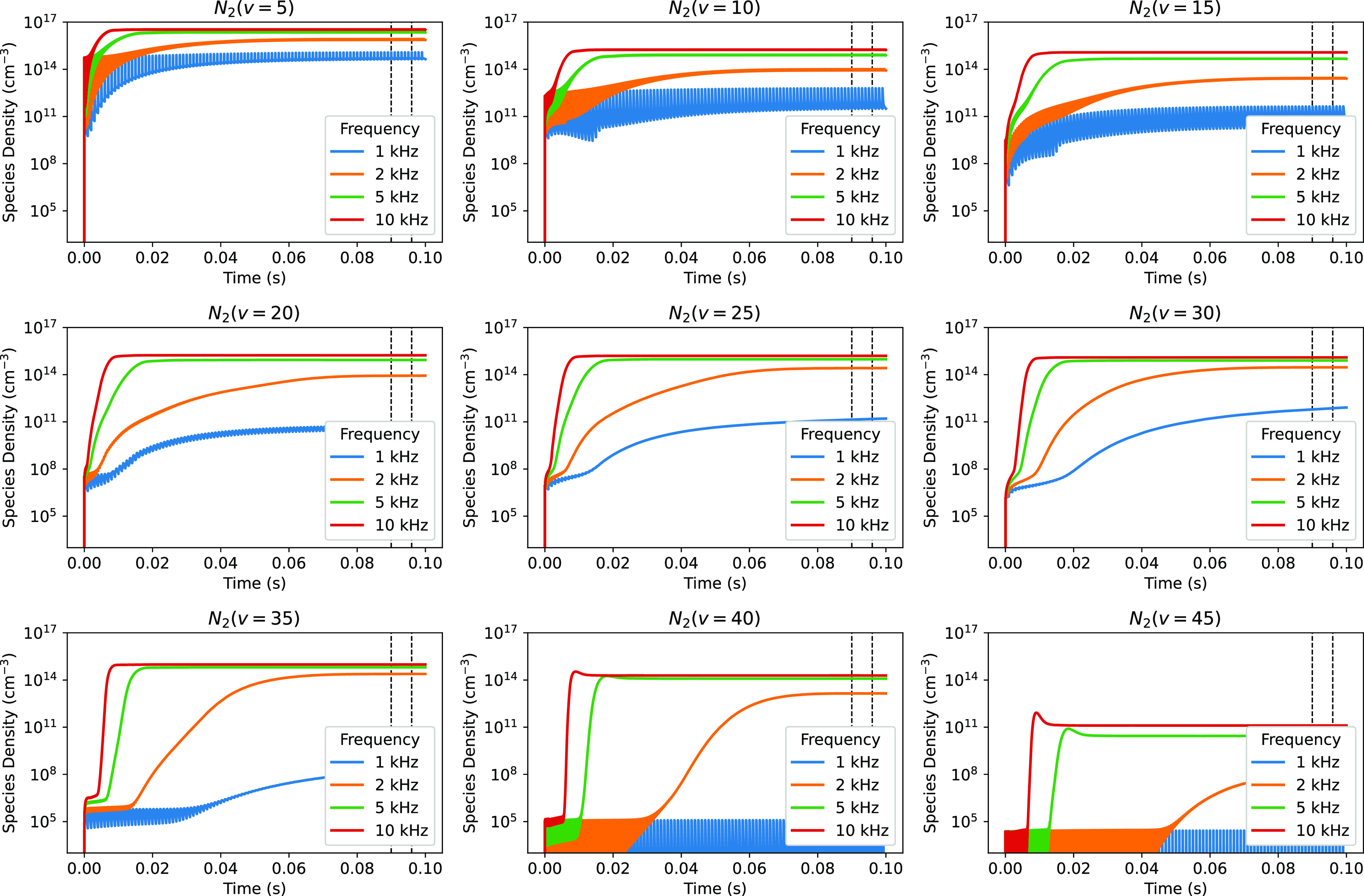
The time evolution of the densities of N$_2(v = 5,10,15,20,25,30,35,40,45)$ as a function of pulse repetition frequency. For each panel, the base case (1 kHz) is indicated by the blue line, showing the lowest densities. The other lines are for the 2 kHz, 5 kHz and 10 kHz cases, which show increasing concentrations with increasing frequency. For all cases, the peak pulse power is 45 W and the pulse width is 10 *µ*s. The vertical dashed black lines signify the time range for averaging the species densities used in figure 9.

Figure 8 shows the densities of N$_2(v = 5,10,15,20,25,30,35,40,45)$ as a function of time, for four different pulse repetition frequencies, 1 kHz, 2 kHz, 5 kHz and 10 kHz. The 1 kHz case is the base case, the same as the 45 W peak pulse power case in figure 4.

When varying the pulse repetition frequency, the trends seen in the different vibrationally excited states are largely consistent with those seen in figure 4 when the peak pulse power is varied. As with the peak power variation:

• There is an overall increase in density over time, for all frequencies and vibrationally excited states up to, and including N$_2(v = 35)$.
• The low vibrational levels (≈N$_2(5\leqslant v \leqslant 15)$) show large fluctuations in density, increasing during the power on-time and decreasing during the power off-time. The medium vibrational levels (≈N$_2(20\leqslant v \leqslant 30)$) show some initial fluctuations in the early stages of the simulation, after which their densities rise more quickly, with less dependence on the power on- and off-times, shown by the fluctuations becoming smaller (relative to the overall density). Finally, the higher vibrational levels (≈N$_2(v \geqslant 35)$) show differing kinetics depending on the pulse repetition frequency. Pulse repetition frequencies higher than the 1 kHz base case show initial species fluctuations (similar to the low vibrational levels), after which the densities rise quickly, and lose dependence on the power on- and off-times. The high vibrational levels are never strongly populated for the base case.
• The species reach similar plateau densities in the 2 kHz, 5 kHz and 10 kHz cases, though the plateau reached in the 2 kHz case is lower than for the higher frequency cases.
• There is potential for achieving the most energy efficient conditions for production of high energy vibrational states (≈$v \geqslant 20$) by alternating periods of pulsing and periods where no power is applied. In both the 10 kHz and 5 kHz cases, the maximum species density is reached by 20 ms into the simulation. Beyond this, there is no further increase in density. However, for the pulsed pulsing situation to achieve the intended results, the pulse frequency must be sufficient to reach the maximum plateau density (5–10 kHz here) and the pulsed period must be sufficient to allow the plateau to be reached (≈20 ms here).

#### 3.4.2. Comparison of frequency and power variation.

**Figure 9.**
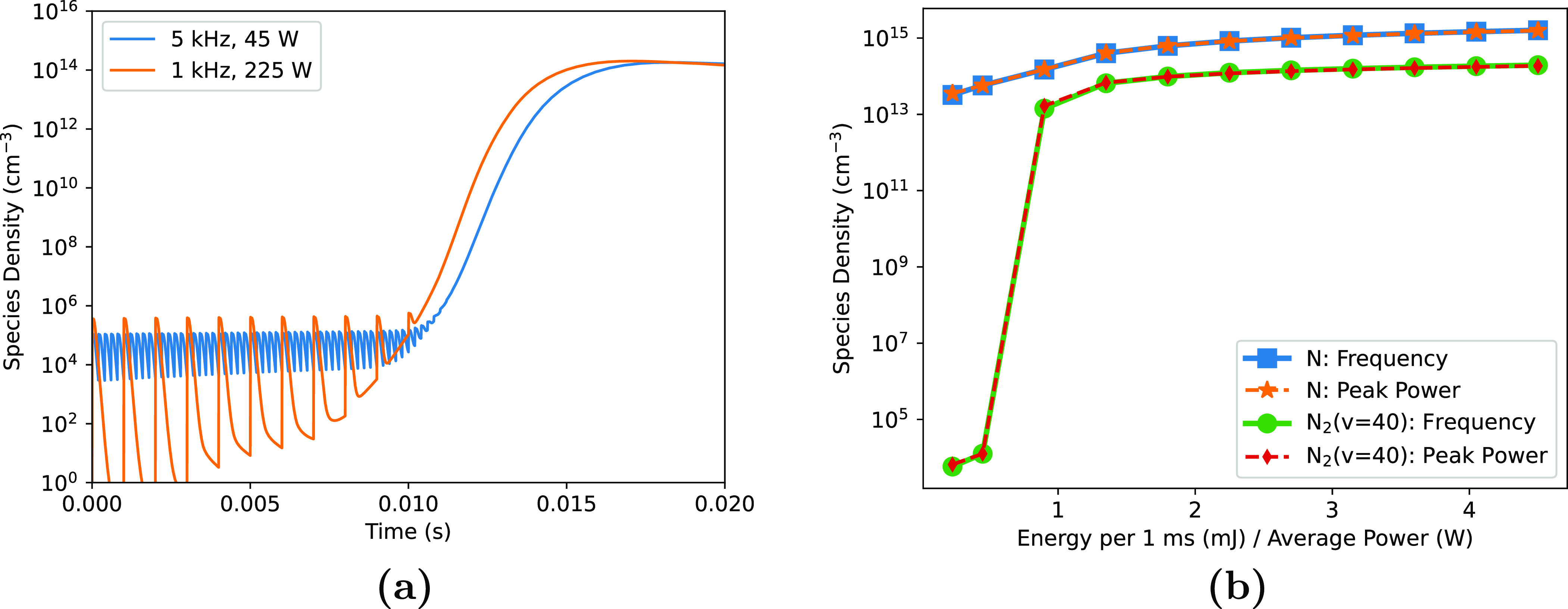
The effects of varying peak pulse power or pulse repetition frequency to deliver a specific energy to the plasma. (a) shows the temporal evolution of N$_2(v = 40)$ in the high power or high frequency cases. (b) shows the average densities of N and N$_2(v = 40)$ as a function of energy deposition, achieved by altering the peak pulse power (- - - -, indicated by ‘Peak Power’ in legend) or the repetition frequency (––––, indicated by ‘Frequency’ in legend). The average densities were calculated over the 90–96 ms period, indicated by the vertical dashed lines in figures 4 and 8.

While the kinetics are largely very similar between the power and frequency variation, there are some subtle differences between the two cases. These relate to the difference in the peak power input during each pulse. This can be seen by comparing equivalent pairs of simulations when the total energy input to the plasma over a given time frame, or the average power deposition, is the same but delivered either through a higher peak pulse power, or a higher pulse frequency, as shown in table 4. For the higher peak pulse power cases (as in the power variation), the higher power results in higher peak electron densities during the pulse, in comparison to the frequency variation, where the peak pulse powers are lower, and the peak electron densities are lower. This means that the short-term kinetics (changes in single pulses) vary between the power and frequency variation cases. However, for the frequency variation, the lower peak electron density is counteracted by the higher pulse frequency and resultant shorter off-time, compared to the power variation data. While over longer timescales, this has little effect (the plateau density appears to be unaffected), it does result in the densities of some species in the high frequency case lagging behind the high power case slightly. An example of this is shown in figure 9(a), where the densities of N$_2(v = 40)$ are shown in equivalent high power or high frequency cases. In both cases, 2.25 mJ of energy are delivered every 1 ms. Here, from about 10 ms, the N$_2(v = 40)$ density rises rapidly in the high power cases, while there is a short time lag ($\lt$1 ms) before the density in the high frequency case follows. This is likely due to the fact that an energy threshold required for the rapid density increase is met at the start of a 1 ms period for the high power case, whereas this threshold is reached later in the higher frequency case where more pulses in the 1 ms period are required. There are also more subtle differences between the different cases, which come about through the different electron energies achieved during the pulses. For example, at the 10 ms point, the electron-impact reaction rate coefficient for $e^- + $N$_2 \rightarrow$ N$_2(v = 40) + e^-$ is $7.4 \times 10^{-19}$ cm^3^ s^−1^ in the 225 W, 1 kHz case, while for the 45 W, 5 kHz case, the same coefficient is $2.5 \times 10^{-19}$ cm^3^ s^−1^. However, these effects are minimal, compared to the effects of the different temporal energy depositions between the frequency and power variations.

**Table 4.** Total energy deposition over a time period of 0.001 s, and average power deposition, for different peak power and pulse frequency combinations used in the variations.

		Power variation	Frequency variation
Energy	Average power	Peak power (Frequency)	Frequency (Peak power)
0.45 mJ	0.45 W	45 W (1 kHz)	1 kHz (45 W)
0.90 mJ	0.90 W	90 W (1 kHz)	2 kHz (45 W)
2.25 mJ	2.25 W	225 W (1 kHz)	5 kHz (45 W)
4.50 mJ	4.50 W	450 W (1 kHz)	10 kHz (45 W)

Despite the differences depicted in figure 9(a), within this range of frequency and power parameters that are typical for DBDs of this type, the final densities of plasma species are largely the same when energy deposition is kept constant. To show the similarities between species densities in the frequency and power variation simulations, figure 9(b) gives the densities of N and N$_2(v = 40)$ averaged over the 90–96 ms time range in each case. Data is shown as a function of energy deposition per 1 ms. This figure shows that for both the representative vibrationally excited state (N$_2(v = 40)$) and non-vibrationally excited state (N), when the energy input is consistent, the densities are almost identical, irrelevant of the peak pulse power or frequency. Other vibrationally excited and non-vibrationally excited species show the same agreement between the two parameter variations, but are not shown here. Overall, this indicates that it is energy deposition, or average power, that are the most important factors for determining the species densities, rather than the ‘shape’ of the power input, at least in the parameter ranges investigated here. A similar conclusion was reported for the density of N in [92].

### 3.5. Role of V–V reactions

Vibrational energy exchange reactions can lead to either an increase, or decrease in vibrational level. Earlier, it was demonstrated that vibrational energy exchange processes become the primary source of higher vibrationally excited states after a certain period of time has passed. Here, the role of V–V reactions will be discussed in more detail.

In general, V–V reactions provide a mechanism for one vibrationally excited state to climb the vibrational ladder through collisions with another vibrationally excited state. The absolute reaction rate of a V–V process depends on the densities of the two collision partners, and the reaction rate coefficient for the process ($k^{\,w-1, w}_{v, v-1}$). The latter is strongly dependent on the vibrational number of the two vibrationally excited states undergoing the collision.

As an example, consider the up-pumping processes for $N_2(w = 36)$ as follows:

\begin{equation*} \mathrm {N}_2(w-1 = 35) + \mathrm {N}_2(v) \rightleftharpoons \mathrm {N}_2(w = 36) + \mathrm {N}_2(v-1). \end{equation*}

**Figure 10.**
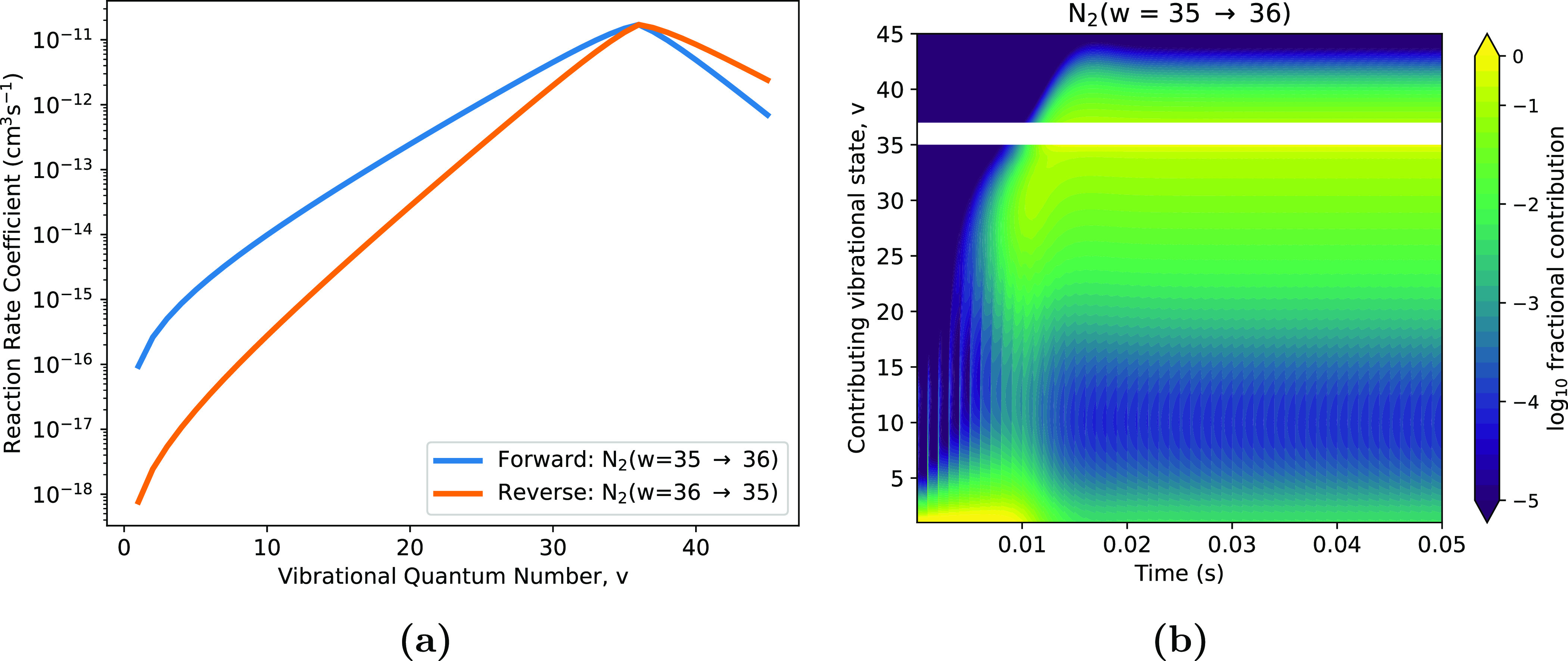
Production of N$_2(w = 36)$ by vibration–vibration (V)–(V) reactions. (a) Shows the forward and reverse V–V reaction rate coefficients for the reaction N$_2(v) + $N$_2(35) \rightarrow $N$_2(v-1) + $N$_2(36)$ at 300 K. (b) Shows the relative contribution of N$_2(v)$ (contributing vibrational state, *y* axis) to the production of N$_2(w = 36)$, as a function of simulation time. Data shown is from the 225 W peak pulse power case, with a repetition frequency of 1 kHz and a pulse width of 10 *µ*s. Values shown are log_10_ of the fractional contribution of N$_2(v)$ to the production of N$_2(w = 36)$ from N$_2(35)$. The reaction N$_2(36) + $N$_2(35) \rightleftharpoons $ N$_2(35) + $N$_2(36)$ is omitted (*v* = *w*, blank area in plot), as this does not result in a net gain of N$_2(w = 36)$.

The forward and reverse reaction rate coefficients at 300 K for (5) are shown in figure 10(a), where it can be seen that the rate coefficient varies by many orders of magnitude, depending on the value of *v*. There are three features of particular importance considering the rates: (a) the forward reaction is favoured over the reverse when *w* > *v*, i.e. when the forward reaction is exothermic to a significant degree; (b) when *v* ≈ *w*, within one or two vibrational levels, then rate coefficients for forward and reverse processes are approximately equal; (c) when *w* < *v*, the reverse reaction is exothermic and is favoured over the forward.

The absolute reaction rate for the reaction given by (5) will depend on the densities of N$_2(v)$ and $k_{v, v-1}^{\,35, 36}$. This means that for N$_2(w = 35 \rightarrow 36)$, the dominant reaction will be the one where the product of [N$_2(v)$] and $k_{v, v-1}^{\,35, 36}$ is greatest, achieved either by a large density of N$_2(v)$, a high $k_{v, v-1}^{\,35, 36}$, or both. To illustrate this, and the up-pumping effects seen in the simulated vibrational states, contour plots showing the relative contribution of different vibrational levels (values of *v*) to (5) are shown in figure 10(b).

In figure 10(b), it can be observed that during the initial stages of the simulation, the production of N$_2(w = 36)$ from N$_2(w = 35)$ is mainly due to the low vibrational states (N$_2(v = 1,2,3\ldots)$). This is the stage where [N$_2(v)$] is high, resulting in a high [N$_2(v)$] and $k_{v, v-1}^{\,35, 36}$ product. However, as time progresses, it can be seen how higher values of *v* become more important for the production of N$_2(w = 36)$ from N$_2(w = 35)$. Between 7 ms and ≈14 ms, N$_2(v = 15-34)$ are the most important reaction partners for N$_2(w = 35)$. Since *v* < *w*, this is indicative of a classical vibrational up-pumping process, where the anharmonicity of the vibrational energy levels results in ladder climbing of the vibrational states. After ≈15 ms, the up-pumping pattern is still seen (there is more contribution from *v* < 35 than *v* > 36), however, there is also a significant production from states of *v* > 36. This is because, as time progresses, the densities of the higher vibrational states increase. Therefore, the reverse processes with lower reaction rate coefficients ($k_{v, v-1}^{\,35, 36} \gt k_{v-1, v}^{\,36, 35}$) can happen more significantly as the density of N$_2(v)$ is sufficient to offset the lower rate coefficient.

**Figure 11.**
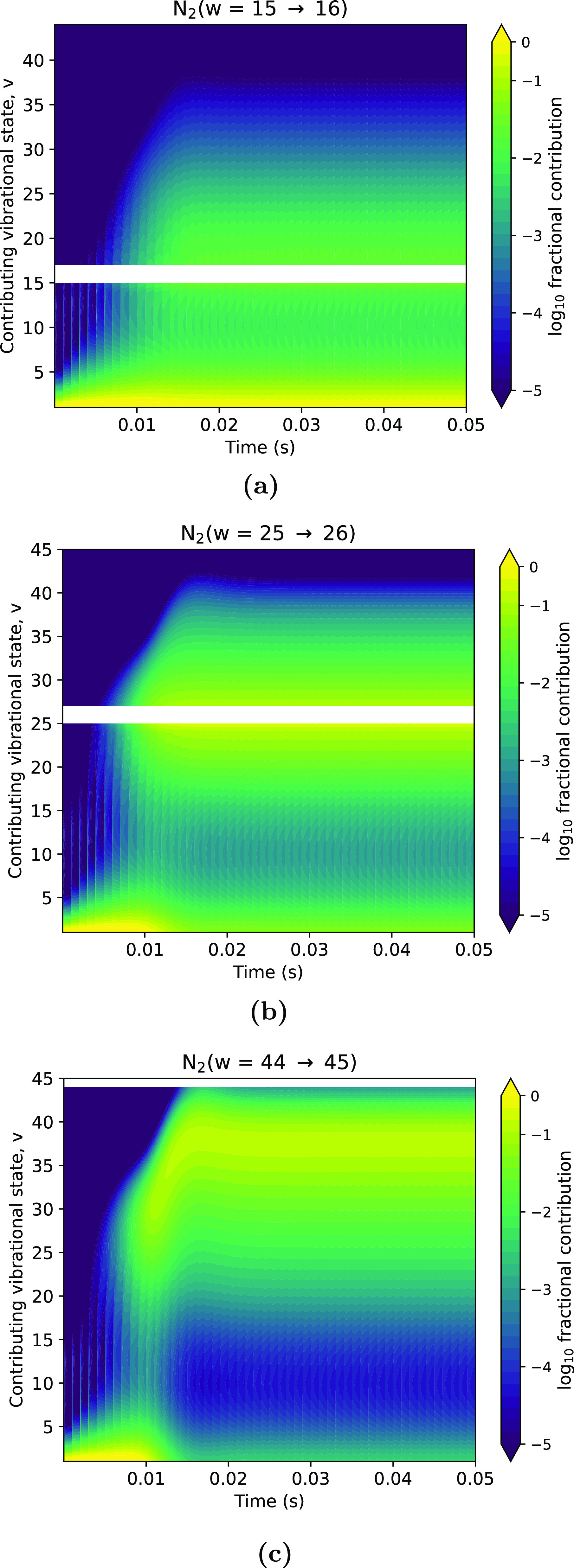
Relative contribution of N$_2(v)$ (contributing vibrational state, *y* axis) to the production of N$_2(w)$, as a function of simulation time. Data from the 225 W peak pulse power case is shown for *w* = 16 (a), *w* = 26 (b) and *w* = 45 (c). Reactions N$_2(v) + $N$_2(w-1)$ where *w* = *v* are omitted (blank area in plot) as these reactions do not result in a net increase of N$_2(w)$ from N$_2(v)$. The pulse repetition is 1 kHz and the pulse width is 10 *µ*s.

Using this type of analysis, it is possible to indicate where this classical V–V up-pumping becomes vital for the vibrational kinetics seen in figures 4 and 8. In figure 11, the contributions of N$_2(v)$ to the processes of N$_2(w-1 \rightarrow w$, where $w = 16, 26, 45$), are shown. For *w* = 16 and *w* = 26 it can be seen that the low vibrational states are still extremely important. For *w* = 16, this importance persists throughout the simulation time, however, for *w* = 26, it drops after the first 15 ms. In both cases, at about 15 ms, extra values for *v* become important for the reaction. Interestingly, the new dominant values of *v* are those that are close to the value of *w*. This is most clearly seen in the case of *w* = 25, where there is a distinctive region of importance of *v* ≈ $20-28$. This is because when *v* ≈ *w* the value of $k_{v, v-1}^{\,w-1, w}$ reaches its maximum value. However, these are not the initial dominant processes for production of N$_2(w)$ as they require the density of N$_2(v)$ to be such that the value of $k_{v, v-1}^{\,w-1, w}$ is the rate limiting factor. For the *w* = 45 case, the up-pumping over time can be clearly seen. After the initial dominant contribution from low values of *v*, the dominant value of *v* increases steadily from *v* ≈ 20 up to *v* ≈ 40. As shown in figures 4 and 8 above, the concentrations of vibrationally excited states rise at different times in the simulations, with the lower states increasing in concentration first, and higher ones increasing in concentration sequentially later. Therefore, the increasing value of *v* contributing to N$_2(v = 45)$ production via V–V processes follows the sequential increase in concentrations of higher vibrational states, which then switches the rate limiting factor from [N$_2(v)$] to $k_{v, v-1}^{\,w-1, w}$.

The full state-to-state consideration of all V–V reactions is highly complex. However, this analysis suggests that the most important reactions for a particular value of *w* are when *v* is small, and when *v* ≈ *w*. Therefore, if the reaction set were to be reduced to only include the V–V reactions where *v* = 1 and when *v* = *w*, this should give a reasonable first-order approximation of the vibrational kinetics. This is in line with the results of VDF calculations for CO_2_ based on the Fokker–Planck approach from Viegas *et al* [95], and may give directions for future simplifications of the very complex coupled vibrational kinetics.

This analysis demonstrates that V–V up-pumping can occur in atmospheric pressure DBD systems, in a similar way to under low pressure conditions where such kinetics are well characterised. This is also in agreement with the works of Colonna *et al* [32], where up-pumping was observed in the afterglow of an atmospheric pressure nitrogen plasma, following a single nanosecond scale pulse. The pulse used in their study is much shorter than the pulse used in these present simulations, and therefore, might explain the difference in timescales for up-pumping to begin. In their study, a single pulse was sufficient to initiate the up-pumping process and the increasing population of the high energy tail of the VDF.

In addition to providing a mechanism for the population of higher vibrational states, V–V processes are also an important vibrational loss mechanism. In fact, in this work, it was seen that for high vibrationally excited states, the dominant destruction mechanisms were V–V processes, while V–T processes were less important, at least for the timescales considered.

### 3.6. The vibrational–electronic (V–E) link

Above, in figures 7 and 9(b) it was seen that increasing the peak power or frequency of the pulses powering the plasma resulted in an increased density of atomic nitrogen, electrons, N$_2(A)$ and N$_2(a^{\prime})$. Closer inspection reveals that increasing the energy deposition/average power appears to result in increased densities, via the population of the VDF.

**Figure 12.**
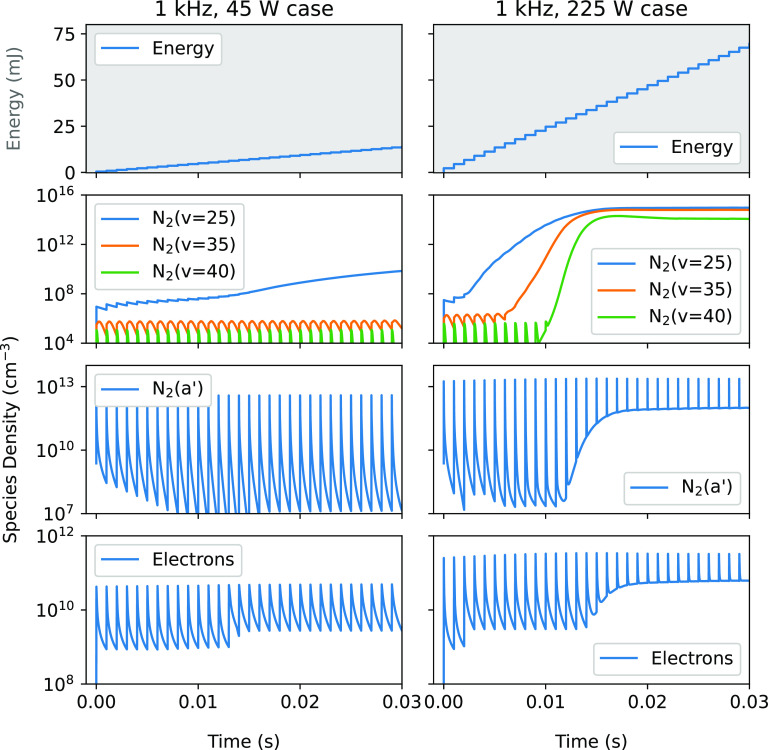
Temporal evolution of energy deposition, vibrationally excited state densities, $N_2(a^{\prime})$ metastable density and electron density, when the peak pulse power is 45 W (left panels) or 225 W (right panels).

In the bottom two panels of figure 12, the densities of electrons and N$_2(a^{\prime})$ as a function of time for the base case (45 W, 1 kHz, left panels), and the 225 W peak power (1 kHz, right panels) case are shown. For each of these species in the base case (left), it can be seen that the fluctuations in species densities are generally very consistent throughout the whole simulation time. However, in the 225 W case (right), the trend seen is quite different. Instead of the generally repeatable density fluctuations, there is a point when the minimum density rises by at least an order of magnitude, resulting in a higher average density. Interestingly, this increase correlates with the time point that the densities of the high vibrational levels begin to rise rapidly. In fact, when looking at the vibrational state densities, metastable density and electron density in the 225 W case, it appears that highly vibrationally excited states are produced, after which there is an increased production of metastables in the power-off times between pulses. Similarly, following the increase in metastable density, there is an increase in the electron density. These features suggest that population of the high energy end of the VDF may be responsible for an increase in average metastable density, and subsequent rise in average electron density.

Analysis of the dominant production mechanisms of the metastable states in this situation reveals that there are two important reactions that transfer energy from the vibrationally excited species to the metastable states. These reactions are:

\begin{align*} \textbf{rxn 51:} \quad \mathrm {N} + \mathrm {N}_2(40 \leqslant v \leqslant 45) &amp; \rightarrow \mathrm {N}(^2D) + \mathrm {N}_2(A) \\ \textbf{rxn 52:} \quad \mathrm {N} + \mathrm {N}_2(39 \leqslant v \leqslant 45) &amp; \rightarrow \mathrm {N} + \mathrm {N}_2(a^{\prime}). \end{align*}

For the production of electrons, alongside electron impact ionisation, Penning ionisation processes can also be important contributors under certain conditions, as follows:

\begin{align*} \textbf{rxn 26:} \quad \mathrm {N}_2(A) + \mathrm {N}_2(a^{\prime}) &amp; \rightarrow \mathrm {N}_4^+ + e^- \\ \textbf{rxn 27:} \quad \mathrm {N}_2(a^{\prime}) + \mathrm {N}_2(a^{\prime}) &amp; \rightarrow \mathrm {N}_4^+ + e^-. \end{align*}

In this study, following the rise in metastable density, the absolute reaction rates of Penning ionisation processes also rise, significantly increasing the electron production in the off-time, and increasing the overall electron density. Therefore, under the conditions studied here, reactions 51 and 52 link the densities and kinetics of vibrationally excited states to those of electronically excited states, which themselves are linked to electron kinetics via reactions 26 and 27. This highlights the strong coupling between vibrationally excited states and the rest of the plasma species.

Reactions 51 and 52 were proposed in [13] to explain the nitrogen pink afterglow, and after performing a variation, the authors concluded that the appropriate rate coefficient for these reactions is $1 \times 10^{-11}$ cm^3^ s^−1^. Given the importance of these reactions in our reaction scheme, and the fact that their rate coefficients do not come from direct experimental measurements, further analysis of their effects is justified. Therefore, a variation of the reaction rate coefficients for these reactions was performed. To do this, the rate coefficient for these reactions was varied between the value of $1 \times 10^{-11}$, determined in [13] and $1 \times 10^{-14}$, a value slightly below the V–Ta rate coefficients for the vibrational levels of $v \gt35$.

**Figure 13.**
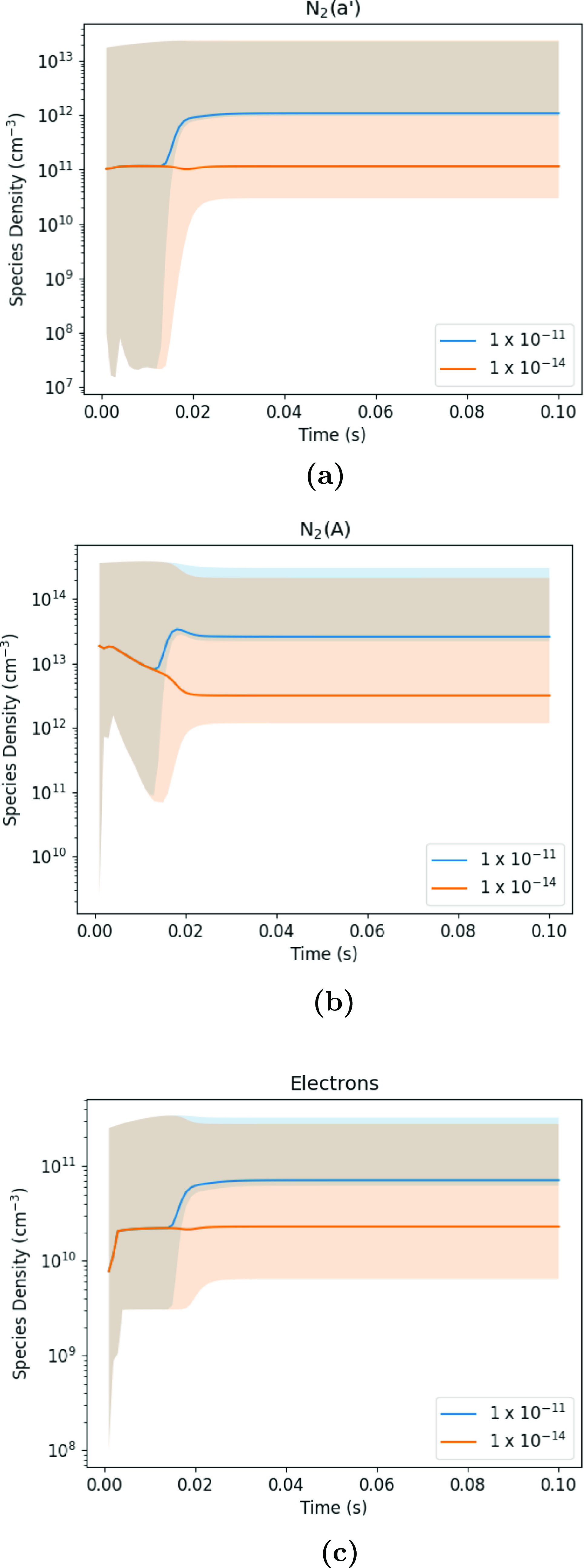
Densities of N$_2(a^{\prime})$ (a), N$_2(A)$ (b) and electrons (c) as a function of time in the 225 W peak pulse power case, when the value of $k_{51/52}$ is set to $1 \times 10^{-11}$ (blue) or $1 \times 10^{-14}$ (orange) cm^3^ s^−1^. Densities plotted are the moving average value of the species, averaged over the previous 1 ms window. The shaded area shows the maximum and minimum density for the species in the window.

In figure 13, the moving average densities of electrons, N$_2(A)$ and N$_2(a^{\prime})$ are shown for the 225 W pulse power case. The blue and orange lines refer to $k_{51/52} = 1 \times 10^{-11}$ cm^3^ s^−1^ and $k_{51/52} = 1 \times 10^{-14}$ cm^3^ s^−1^, respectively. The shaded areas show the moving minimum and maximum densities in each of the cases. At the start of the simulation, the densities are the same, irrespective of $k_{51/52}$. However, at a certain time point (≈12 ms), the reaction rate coefficient starts to have an effect. When $k_{51/52} = 1 \times 10^{-11}$ cm^3^ s^−1^, there is a significant increase in the average species density for the remainder of the simulation time. This is achieved by an increase in the minimum density reached in the off-times between pulses (shown by the blue shaded areas). This increase in the density is not seen when the value of $k_{51/52}$ is low at $1 \times 10^{-14}$ cm^3^ s^−1^. Instead, the density either remains constant for the whole simulation period (electrons and N$_2(a^{\prime})$) or decreases, then remains constant (N$_2(A)$).

**Figure 14.**
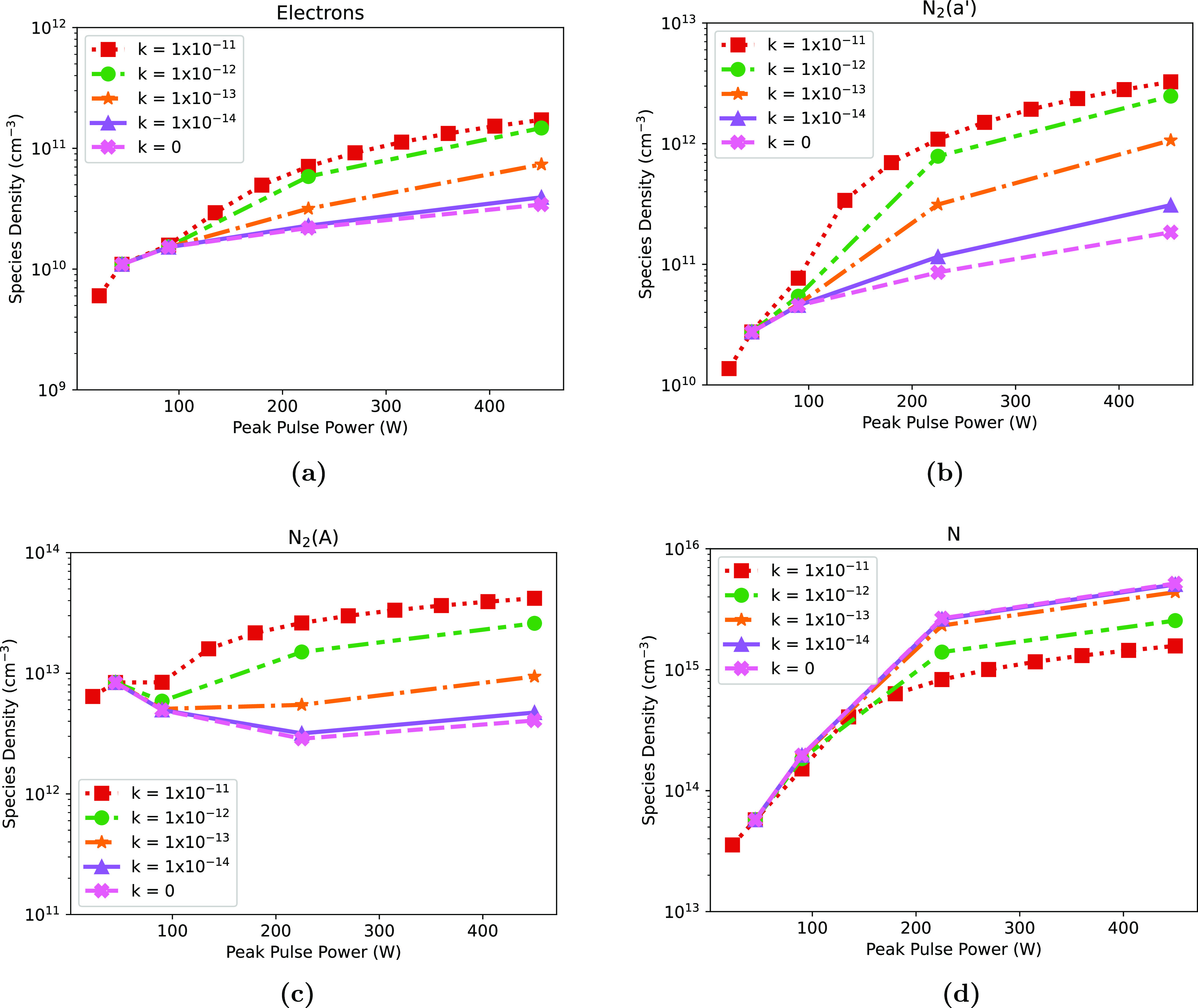
Average densities of electrons (a), N$_{2}(a^{\prime})$ (b), N$_2(A)$ (c) and atomic nitrogen (d) in the equilibrium phase of the simulation, for different values of $k_{51/52}$. The average density is calculated over the time period 90–96 ms. The pulse repetition frequency and pulse width are 1 kHz and 10 *µ*s, respectively.

In figure 14, a power variation is performed once more, for a series of values of $k_{51/52}$. Here, the average densities once the equilibrium phase has been reached (90–96 ms) are taken for each value of $k_{51/52}$, for each of the pulse powers. At low powers, the reaction rate coefficient makes little difference. This is due to the fact that in these low powers, the densities of the high vibrational levels remain low, therefore, these reactions occur at a low rate, even when the reaction rate coefficient is large. As the power is increased, the difference in species densities increases when $k_{51/52}$ is varied. This is due to the VDF becoming increasingly populated as the power increases, therefore, more vibrational energy can be converted into other species by reactions 51 and 52.

For N$_2(a^{\prime})$, the effect of increasing the rate coefficients of $k_{51/52}$ is straightforward. With increasing reaction rate coefficient, the density of N$_2(a^{\prime})$ rises for a constant power. This is due to an increased absolute rate of the N$_2(a^{\prime})$ producing reaction 52. Increasing the power also serves to increase the density of N$_2(a^{\prime})$ as discussed earlier.

For N$_2(A)$, increasing the reaction rate coefficient also results in an increase in N$_2(A)$ density for a given power, due to the increase in absolute rate of reaction 51. However, for an increase in power to show a clear increase in species density, the reaction rate coefficient for reaction 51 must be at least $1 \times 10^{-12}$ cm^3^ s^−1^.

For the electrons, once again, an increase in $k_{51/52}$ results in an increased density at each power, and an increase in density for increasing power and constant values for $k_{51/52}$. The rise in electron density is as a result of an increase in Penning ionisation (reactions 26 and 27), allowed through the increasing densities of N$_2(a^{\prime})$ and N$_2(A)$.

Atomic nitrogen shows the opposite relationship with $k_{51/52}$, compared to the electrons and metastables. Here, for an increasing value of $k_{51/52}$, at a given power, the density of N decreases. This is due the vibrational state kinetics. When $k_{51/52}$ is low, the vibrational energy is not transferred into the metastable states (N$_2(a^{\prime}/A)$), and instead, the absolute reaction rate for vibrational dissociation processes are approximately an order of magnitude higher when $k_{51/52}$ is lowered from $1 \times 10^{-11}$ to $1 \times 10^{-14}$ cm^3^ s^−1^. Specifically, the affected reactions are as follows:

\begin{align*} \textbf{rxn 43:} &amp; \quad 2\mathrm {N}_2(14\leqslant v \leqslant 25) \rightarrow \mathrm {N}_2 + \mathrm {N} + \mathrm {N} \\[6pt] \textbf{rxn 45:} &amp; \quad \mathrm {N}_2(43 \leqslant v \leqslant45) + \mathrm {N}_2 \rightarrow \mathrm {N}_2 + \mathrm {N} + \mathrm {N}. \end{align*}

From this result, the importance of $k_{51/52}$ is highlighted. It appears that if the coupling between the vibrational and electronic states is increased (via higher values of $k_{51/52}$), the efficiency of N production is decreased. Since N production may be one of the desired applications for pure N_2_ plasmas, accurate simulations of N densities and kinetics are valuable, and in order to achieve this, measurements or further theoretical studies of the value of $k_{51/52}$ would be beneficial.

## 4. Discussion and conclusions

Many applications for nitrogen-based plasmas rely on the formation of N$_2(v)$ species in order to aid the dissociation of N_2_, or aid the formation of NO/NO_*x*_ species via the Zel’dovich reaction. This is the case when LTPs are used for nitrogen fixation purposes [5, 31, 46], or for biomedical applications where NO may be play a role as a therapeutic [17, 18]. Therefore, it could be advantageous to be able to tailor plasma chemistry to efficiently produce N$_2(v)$ species.

This work has sought to outline some of the phenomena related to vibrational and electronic excitation in nitrogen discharges at atmospheric pressure under experimentally relevant operating conditions, with a focus on repetitively pulsed sources that are of interest for a variety of applications. These discharges typically have an average power of a few W, and pulse repetition frequencies in the range of 1–10 kHz. The 0-dimensional plasma chemical-kinetics simulations carried out here have predicted that under these conditions, highly vibrationally excited states of N_2_ can become significantly populated, and the extent of the population of the VDF can be influenced by the peak pulse power and the pulse repetition frequency.

When altering the peak pulse power, the finest control of the VDF can be achieved in the region between 70–90 W peak pulse power. In this range, small changes to the peak pulse power result in large rises in the densities of the high vibrational states, which may be of benefit when seeking to control the energy efficiency of species produced via vibrational states. However, once the vibrational states have reached their plateau densities, further increases in energy do not appear to have a significant effect on the their densities or kinetics. Therefore, when the pulse repetition frequency is 1 kHz, peak pulse powers of just greater than 90 W seem to provide the most energy efficient production of vibrational states.

At the point that the high levels of the VDF ($v \geqslant 39$) are significantly populated, energy transfer processes (reactions 51 and 52) that link the densities of vibrational states to those of electronically excited states can have an important effect on the overall chemical kinetics. Via these reactions, vibrational energy is transferred to the N$_2(A)$ and N$_2(a^{\prime})$ metastable species, then, as the metastable densities rise, the rate of Penning ionisation rises, ultimately resulting in an increase in the electron density. As a result of these V–E linking reactions, the sensitive control of non-vibrationally excited species is achieved in the slightly higher power ranges (≈90–200 W peak pulse power), as the transfer of energy from the vibrational states to the electronically excited states becomes important with rising N$_2(v)$ densities.

The limited sensitivity analysis performed in the present work suggests that, under the conditions studied, a reaction rate coefficient for the V–E linking reactions (reactions 51 and 52) of at least $1 \times 10^{-12}$ cm^3^ s^−1^ is required for vibrationally excited states to be able to influence the wider plasma chemistry. These reactions not only control absolute densities of metastable species, but their presence in the reaction scheme with sufficiently high rate coefficients can alter the trends seen during a power variation. This is particularly true for the density of the N$_2(A)$ metastable state. In a similar way to the work of [13] at low pressure, experimental quantification of the N$_2(A)$ densities under the atmospheric pressure conditions studied in this work would be particularly useful to more fully understand the strength of this link and give additional insight into the value of their reaction rate coefficients.

For repetitively pulsed plasmas, it is important to consider both the power on- and off-times that occur with each pulse cycle. During the power on-time, electron processes are particularly important for the production of species. However, during the power off-time, other reactions begin to have significance and raise average species densities, highlighting the importance of including chemistry that accounts for both the kinetics in active discharges (power on-time) and post-discharges (power off-time). Reactions occurring during the power off-time usually involve vibrationally excited states, which lead to the production of species such as higher vibrational states (via V–V processes), atomic nitrogen (reactions 43–45), metastables N$_2(A/a^{^{\prime}})$ (reactions 51 and 52) and electrons (Penning ionisation, reactions 26 and 27). The chemistry occurring during the power off-time then influences the baseline densities occurring in the power on-time, and serve to raise the overall average species densities. It is, therefore, important that such reactions should be included in chemical kinetics simulations of repetitively pulsed plasmas.

The practical importance of being able to control the population of high level vibrationally excited nitrogen species is seen when considering the large amounts of energy that they can carry, which could influence any treated surface or substrate. For example, the simulations carried out here predict that an order of magnitude increase in peak pulse power gives rise to an increase in the equilibrium densities of N$_2(v \geqslant 35)$ of 5–8 orders of magnitude. Since these species carry upwards of 7.9 eV per molecule [10], this relatively small change in energy input to the plasma could have a significant effect on the flux of high energy species reaching a target. Therefore, the understanding of the connection between operating parameters and the resultant vibrationally excited state populations would be beneficial to all areas where atmospheric pressure LTPs are used, for example for technological, agricultural or biomedical applications.

This study also indicates that, within the range of parameters studied, the mechanism by which energy is input to the plasma system, either through an increased peak pulse power or an increased pulse repetition frequency, has little effect on species densities. Instead, the total amount of energy deposited/the average power deposition is more important than the peak power or repetition frequency of the pulses, individually. In principle, this allows some freedom for experimental systems to be able to alter the plasma chemistry using the simplest operating conditions available. For example, altering the pulse repetition frequency is often easier than increasing the peak pulse power.

In summary, the present work investigates kinetics of excited states in nitrogen discharges at atmospheric pressure, across a range of parameters typical of those used in DBD sources. Simulations presented here show that the population of highly excited vibrational states can be affected strongly over a relatively small operating parameter range. This has implications both for the fundamental study of DBD plasmas, but also for applications for these systems. These high level vibrational states can carry significant amounts of energy, and contribute to the overall plasma chemistry, including the kinetics of electronically excited nitrogen molecules, and have the potential to impact on any treated surface or substrate during applications. Future work should be performed to further validate the species densities and trends predicted by these simulations, and to help inform the choice of certain reaction rate coefficients and wall loss parameters.

## Data Availability

Data underpinning the figures in this article will be made freely available in the Research Data Repository of the Research Department ‘Plasmas with Complex Interactions’ at the Ruhr University Bochum under the following URI: https://rdpcidat.rub.de/node/570.

## Appendix A V–V reaction rate coefficients

Collisions between vibrational states (V–V reactions, reactions 58) are typically described in the following form:

\begin{equation*} \mathrm {N}_2(v) + \mathrm {N}_2(w-1) \rightarrow \mathrm {N}_2(v-1) + \mathrm {N}_2(w). \\ \end{equation*}

The rate coefficient is given by k$_{v, v-1}^{\,w-1,w}$. As described in [21], the magnitudes of the rate coefficients for these processes, which depend on the specific values of *v* and *w*, can be determined by a number of different approaches. A commonly used approach is the Schwartz–Slawsky–Herzfeld (SSH) theory, which can be implemented in various forms. Here, we use the following form, where the full matrix of V–V reaction rate coefficients for all values of *w* and *v* is calculated using the following expressions:

When $w \leqslant v$:

\begin{equation*} k_{v, v-1}^{\,w-1,w} = \left(\frac{v}{1-\chi_{e}^{\mathrm {N}_2} v}\right) \left(\frac{w}{1-\chi_{e}^{\mathrm {N}_2} w}\right) F(Y^{\,w-1,w}_{v,v-1}) k^{\,0,1}_{1,0} . \end{equation*}

When *w* > *v*:

\begin{align*} k_{v, v-1}^{\,w-1,w} = &amp; \left(\frac{v}{1-\chi_{e}^{\mathrm {N}_2} v}\right) \left(\frac{w}{1-\chi_{e}^{\mathrm {N}_2} w}\right) F\left(Y^{\,w-1,w}_{v,v-1}\right) k^{\,0,1}_{1,0} \nonumber \\ &amp; \times \textrm{exp}\left[\frac{\Delta E_{v, v-1}^{\,w-1,w}}{k_\mathrm {B} T_g}\right]. \end{align*}

These expressions are based on the work of Loureiro and Ferreira [70], with the normalisation of the reaction rate coefficient $k_{v, v-1}^{\,w-1,w}$ to $k^{\,0,1}_{1,0}$ taken from the more recent review of Guerra *et al* [21]. The normalisation term used in equations (A.1) and (A.2) is given by:

\begin{equation*} k^{\,0,1}_{1,0} (\textrm{cm}^3\,\textrm{s}^{-1}) = \frac{6.35 \times 10^{-17}T_g^{\,3/2}}{\zeta} \end{equation*}

where

\begin{align*} \zeta &amp;= 39.0625 - 1.5625\textrm{max(v,w)}, \quad \textrm{when max(v, w)} \lt 10, \nonumber \\ &amp;\quad 25.2 + 24.1\bigg(\frac{\textrm{max[v,w]}-10}{30}\bigg)^3, \quad \textrm{when max(v, w)} \geqslant 10. \end{align*}

The adiabaticity factor, *F*, is approximated as per [21]:

\begin{align*} F(Y) &amp;= \frac{1}{2}\bigg[3-\textrm{exp}\bigg(-\frac{2Y}{3}\bigg)\bigg]\textrm{exp}\bigg(-\frac{2Y}{3}\bigg), \quad 0 \leqslant Y \leqslant 20, \nonumber \\ &amp;\quad 8\bigg(\frac{\pi}{3}\bigg)^{1/2}Y^{7/3} \textrm{exp}(-3Y^{2/3}), \quad Y \gt 20 \end{align*}

with *Y* given by:

\begin{equation*} Y^{\,w-1, w}_{v, v-1} = \frac{\pi L_{\mathrm {N_2-N_2}}}{\hbar}\bigg(\frac{\mu}{2k_\mathrm {B}T_g}\bigg)^{1/2} \Delta E^{\,w-1, w}_{v, v-1}. \end{equation*}

The constants for each of the equations are given in table A1. In the present study, the gas temperature is assumed to be 300 K.

**Table A1.** Constants for equations to calculate V–V and V–Tm reaction rate coefficients in nitrogen. Values are taken from [52].

Constant	Value	Description
$\chi_e^{\mathrm {N}_2}$	$6.073 \times 10^{-3}$	Morse’s operator parameter
*h*	$ 6.63 \times 10^{-34}$ m^2^ kg s^−1^	Planck’s constant
$\hbar$	$1.055 \times 10^{-34}$ m^2^ kg s^−1^	$\hbar \equiv \frac{h}{2\pi} $
$\omega_{\mathrm {N}_2}$	$4.443 \times 10^{14}$ s^−1^	Morse’s operator parameter
$k_\mathrm {B}$	$1.38 \times 10^{-23}$ m^2^ kg s^−2^ K^−1^	Boltzmann’s constant
$L_{\mathrm {N_2-N_2}}$	$2 \times 10^{-11}$ m	Range of intermolecular forces in N_2_–N_2_ collisions
*M*	$4.650 \times 10^{-26}$ kg	Mass of nitrogen molecule
*µ*	$2.325 \times 10^{-26}$ kg	Reduced mass of nitrogen molecule
$\Delta E^{\,w-1, w}_{v, v-1} $	$\hbar \omega_{N_2}2\chi_e^{\mathrm {N}_2} |w-v|$	Energy difference with transition between level
		*v* to *v* − 1, and *w* − 1 to *w*
$\Delta E_{v, v-1} $	$ \hbar \omega_{\mathrm {N}_2}(1-2\chi_e^{\mathrm {N}_2}v) $	Energy difference between *v* and *v* − 1

Using the above expressions and constants gives rate coefficients in good agreement with the semi-classical calculations of Billing and coworkers [96].

## Appendix B V–Tm reaction rate coefficients

For V–Tm reaction rates (reactions 59), the following equations from [52] are used, with the gas temperature assumed to be 300 K. Equation (B.1) gives the expression for the forwards reaction rate coefficient, and (B.2) gives the rate for the reverse reaction rate coefficient. For V–Tm collisions, only single quantum transitions were considered

\begin{align*} k_{v, v-1} = v\bigg(\frac{1 - \chi_e^{\mathrm {N}_2}}{1 - \chi_e^{\mathrm {N}_2}v}\bigg) \frac{F(Y_{v, v-1})}{F(Y_{1,0})} P_{1,0} \end{align*}

\begin{align*} k_{v-1,v} = K_{v, v-1} \text{ exp} \bigg(-\frac{\Delta E_{v, v-1}}{k_\mathrm {B} T_g}\bigg) \end{align*}

\begin{align*} Y_{v, v-1} = \frac{\pi L_{\mathrm {N_2-N_2}}}{\hbar}\bigg(\frac{\mu}{2k_\mathrm {B}T_g}\bigg)^\frac{1}{2} \Delta E_{v, v-1} \end{align*}

where the *F*(*Y*) term uses equation (A.5) and the normalisation is as follows:

\begin{align*} P_{1,0}(\textrm{cm}^3\,\textrm{s}^{-1}) = \frac{1.07 \times 10^{-12}T_g^{\,3/2}}{0.2772 T_g - 80.32 + 35.5(\frac{v-1}{39})^{\,0.8}} F(Y_{1,0}). \end{align*}

## Appendix C V–Ta reaction rate coefficients

V–Ta reaction rates are calculated from equation (C.1), found in [9, 21]. The V–Ta process can either be a reactive or non-reactive process. In the reactive process, the molecule reactant becomes the atomic product and the atom combines with the remaining atom, at a vibrational level one less than the original vibrationally excited molecule. However, in the non-reactive process, the molecule remains molecular and just drops down a vibrational level [20]. The constants used in equation (C.1) are different for these two instances, and the total V–Ta rate is the sum of the two processes (reactions 60). There is also a threshold vibrational level, $v_{\mathrm {thresh}}$, above which the V–Ta processes can occur.

\begin{equation*} k^{\mathrm {N}_2-\mathrm {N}}_{v, v-1} = A_0 \times \textrm{exp}\bigg(-\frac{A_1}{v} + \frac{A_2}{v^2}\bigg) \end{equation*}

Where $k^{\mathrm {N}_2-\mathrm {N}}_{v, v-1}$ is the rate coefficient (in cm^3^ s^−1^) for the de-excitation of N$_2(v)$ to N$_2(v-1)$ upon collision with atomic nitrogen, *v* is the vibrational state, and *A*_0_, *A*_1_ and *A*_2_ are constants given in table C1. Multi-quantum transitions are highly likely in V–Ta collisions, and the rate coefficient is assumed to be the same for changes of up to five vibrational levels [9, 52, 71].

**Table C1.** Constants used to in the equation for calculating V–Ta reaction rate coefficients in nitrogen [21]. *T*_*g*_ is gas temperature in K, and is taken to be 300 K in all calculations of reaction rate coefficients. *A*_0_ is given in 10^−10^ cm^3^ s^1^.

	Reactive	Non-reactive
*A*_0_	$0.2 \times 2.21 \times 10^4 / T_g^{1.43}$	$0.2 \times 9.24 \times 10^4 /T_g^{1.63} $
*A*_1_	$3.21 \times 10^4 / T_g^{\,0.80}$	$ 1.82 \times 10^4 /T_g^{\,0.70} $
*A*_2_	$2.50 \times 10^5 / T_g^{1.04}$	$ 9.89 \times 10^3 /T_g^{\,0.44} $
$v_{\mathrm {thresh}}$	7	9
